# Supplementary material for: Differential quantification of alternative splicing events on spliced pangenome graphs
Source: PLoS Comput Biol. 2024 Dec 9;20(12):e1012665. doi: 10.1371/journal.pcbi.1012665 (PMC11658704; doi:10.1371/journal.pcbi.1012665)
Supplement: S1 Text — Appendices, Supplementary Figures, and Supplementary Tables. Appendix A, “Spliced pangenome construction and alignment”. Appendix B, “Details on the simulation settings”. Appendix C, “RT-PCR truthset filtering”. Fig A: novel events definitions on annotated spliced pangenome. Fig B: results on simulated data from Drosophila Melanogaster (novel events, default parameters). Fig C: results on simulated data from Drosophila Melanogaster (annotated events, w = 5 for pantas). Fig D: results on simulated data from Drosophila Melanogaster (novel events, w = 5 for pantas). Fig E: results on real data from Drosophila Melanogaster. Fig F: results on real data from Drosophila Melanogaster (all events). Fig G: results on real data from Human (all events). Table A: number of simulated events from Drosophila Melanogaster. Table B: full results on simulated data from Drosophila Melanogaster (annotated events). Table C: full results on simulated data from Drosophila Melanogaster (novel events). Table D: events count for Drosophila Melanogaster experiments on real data. Table E: events count for Drosophila Melanogaster experiments on real data (all events). Table F: efficiency results on real data from Drosophila Melanogaster. Table G: efficiency results on real Human data. (PDF) [file pcbi.1012665.s001.pdf]

Differential quantification of alternative splicing events on spliced pangenome graphs

Supplementary Material

Simone Ciccolella, Davide Cozzi, Gianluca Della Vedova, Stephen Njuguna Kuria, Paola Bonizzoni, and Luca Denti

Appendix A Spliced pangenome construction and alignment

`pantas` requires as input an annotated spliced pangenomes (in GFA format) and RNA-Seq alignments to this structure (in GAF format). However, since these files are not always readily available, we implemented a custom procedure based on the `vg` toolkit [GSN+18] to prepare these inputs. We decided to use `vg` since it is the only framework that currently support spliced alignments to a pangenome. In order to have full control of each step of its construction and be able to annotate the graph with additional information needed for AS events discovery (hence making an *annotated spliced pangenome*), we opted for manually constructing and indexing the graph. Indeed, although `vg` allows a user to automatically build an index via the `autoindex` utility, it does not allow to easily retain information on transcripts and splice junctions, that are needed for correct AS event detection. As suggested by `vg` authors, our procedure starts by chunking the input files (reference genome, gene annotation, and variations) by chromosomes and builds one graph per chromosome. It uses `vg construct` to build a pangenome from the reference chromosome and the set of variations and then it augments the graph with transcripts and splice junctions information using the `vg rna` utility. The resulting spliced pangenome contains new edges representing the annotated splice junctions but does not contain the haplotype-specific transcripts yet. Indeed, as suggested by `vg` authors, to maintain the construction as efficient as possible, it is more convenient to first build the spliced pangenome, extract the full haplotypes from the graph (using `vg gbwt`), and finally rerun the `vg rna` utility to project the reference transcripts against the haplotypes, thus obtaining the set of haplotype-aware transcripts. This is the methodology implemented in `pantas`. Once a spliced pangenome with haplotype-aware transcripts is available, `pantas` proceeds to simplify it to remove complex regions and improve the feasibility of the indexing step. `pantas` provides three different simplification steps. The default simplification consists in pruning only the intergenic and intronic regions of the graph. This is done by including all haplotype-aware trascripts and then using the `vg prune` utility asking to restore all paths (i.e., all transcripts) in the graph (setting the optional argument `--restore-paths`). A more aggressive simplification consists in forcing the inclusion of only the reference transcripts. Similarly to the default simplification, this is done by including the reference transcripts in the graph and then running the `vg prune` utility. With this second alternative, exonic portions of the genes could be simplified if too complex. The last simplification consists in removing all intergenic regions and thus retaining only the genic loci. The final graph is a set of connected components, each one representing a gene locus. We notice that a gene locus can potentially include multiple genes in the case of overlapping genes. The choice of which simplification to perform is use case dependent and needs to be decided by the user. The default simplification should provide the most accurate results (since all haplotype-aware transcripts are retained) but indexing the resulting graph will result very computationally expensive. On the other hand, the more aggressive simplification produces a graph that is easier to index, at the expense of potentially lower precision in calling haplotype-aware events (since some haplotype-aware transcripts may be removed and then not annotated). Finally, the third simplification provides the smallest graph while maintaining full information on haplotype-aware transcripts but it is especially useful when the user is interest in analyzing a small panel of genes (as it is the case in our experimental evaluation on real data).

After simplifying the graph, our procedure proceeds to annotate the vertices and the edges of the graph with additional information. Namely, it tags each vertex belonging to an exon with the name of the haplotype-aware transcript it belongs to and the exon number (along that transcript) whereas each edge representing a splice junction with the number of the two linked exons and the haplotype-aware transcript. The annotation iterates over the haplotype-aware transcripts retained in the graph and tag its vertices.

Every time an edge is not present in the corresponding haplotype, it is by definition a splice junction and is tagged as such. We note that vertices and junctions can be shared by more transcripts and therefore annotated with multiple tags (i.e., a set). This information is a valuable information needed for AS events inference from graph alignment. These chromosome-level annotated spliced pangenome graphs are then merged together (without modifying the vertex id space) and the GCSA2 index of the resulting graph is built using the `vg index` utility. We note that spliced pangenome construction and indexing is a one-time expense and the index can be reused for any number of RNA-Seq datasets.

Once an annotated spliced pangenome index is available, the input RNA-Seq dataset can be aligned to it using the `mpmap` aligner [SEN+23]. This aligner was specifically introduced to align RNA-Seq to spliced pangenomes and to this aim it can align over novel splice junctions, that are edges not present in the input graph. This feature is essential for inferring novel AS events. The pipeline to prepare the input for `pantas` is provided alongside the main `pantas` codebase at `github.com/algolab/pantas`.

Appendix B Details on the simulation settings

To evaluate `pantas` on simulated data, we considered the Drosophila Melanogaster reference and gene annotation (FlyBase [TGS+19], r6.51) and the Drosophila Genetic Reference Panel (v2) [MRS+12]. To avoid unnecessary complexity from our exploratory analysis on simulated data, we removed all overlapping genes from the gene annotation and we considered only SNPs (Single Nucleotide Polymorphisms) falling in the resulting set of genes with minimum count and frequency for non-reference allele equal to 1 and 0.01, respectively. The resulting reference panel consists of 2 311 679 SNPs and 205 inbred lines (samples). After randomly selecting one sample from the reference panel, we created its genomic sequences (both haplotypes) using `bcftools consensus` [DBL+21] and then we simulated a RNA-Seq dataset (2 conditions, 1 replicate) using `asimulator` [MTF+21]. We generated 25 000 000 read pairs and we enabled the simulation of exon skipping, alternative acceptor, alternative donor, and intron retention events (with a maximum of 1 event per gene). `asimulator` works by selecting a transcript as *template* transcript and, starting from this, it introduces AS events by altering the template and creating additional *alternate* transcripts. In doing so, it creates a new annotation containing all the generated transcripts. In our analysis, we considered this annotation as input for the tested tools and not the original gene annotation since `asimulator` may merge different transcripts in a single template transcript, thus creating chimeric transcripts not present in the input annotation. Starting from the `asimulator` annotation, which contains all transcripts used to simulate reads (template transcript and alternate transcripts), we also created a reduced annotation containing the template transcript only. This reduced annotation is used to simulate a poor annotation and test the accuracy of tools in calling novel AS events. Based on this data generation, we evaluated the accuracy of `pantas`, `rMATS` [SPL+14], `SUPPA2` [TEH+18], and `whippet` [SWWB+18]) in terms of Precision, Recall, and F1-Measure computed by comparing the events reported by `asimulator` with the events reported by the tools. In the annotated setting, we considered an event to be a *True Positive* if the reported splice junctions are correct with respect to the junctions reported by the simulator. In the novel setting, instead, due to the higher complexity of correctly detecting the novel splice junction, we considered an event to be a *True Positive* if at least one splice junction involved in the event is the correct one. Given the noise present in the truth created by `asimulator` (i.e., events barely supported and events with very low differential expression), we retained only those events with $|\Delta\psi| \geq 0.05$ and we created several truth sets filtering out all events supported by less than $\mathcal{W}$ reads, with $\mathcal{W} \in \{1, 3, 5, 10, 20\}$. For instance, with $\mathcal{W} = 3$, we filtered out all events reported by `asimulator` supported by less than ($<$) 3 reads. Therefore, a bigger $\mathcal{W}$ value results in a smaller sets of highly supported events, as also reported in Suppl. Table A. We used this filtered sets to compute the true positives and false negatives of the tools while we considered the original unfiltered truth set for computing their false negatives. In such a way, we managed to penalize the tools for identifying false events with low support without penalizing them for calling true events supported by fewer reads (less reads than $\mathcal{W}$).

Appendix C RT-PCR truthset filtering

The original RT-PCR truthset provided by [TEH$^+$18] consists of 83 events on 82 genes. However, we note that the original RT-PCR validated events are reported on the `hg19` reference genome. We then used the `liftover` utility to convert their coordinates to the newer `hg38`. However, for three of the 83 events, the remapped coordinates disagreed with the gene annotation (due to a change in start/end position of the skipped exons). We then filtered these three events out and focused our analysis on the resulting set of 80 events. From this set of RT-PCR events, we also removed all events reported with low differential change between the two conditions and we considered all events with $|\Delta\psi| \geq 0.05$. This filter removed 3 additional events from the truth set. The resulting truthset consists in 77 RT-PCR validated events.

Bibliography

DBL$^+$21. Petr Danecek, James K Bonfield, Jennifer Liddle, John Marshall, Valeriu Ohan, Martin O Pollard, Andrew Whitwham, Thomas Keane, Shane A McCarthy, Robert M Davies, et al. Twelve years of samtools and bcftools. *Gigascience*, 10(2):giab008, 2021.

GSN$^+$18. Erik Garrison, Jouni Sirén, Adam M Novak, Glenn Hickey, Jordan M Eizenga, Eric T Dawson, William Jones, Shilpa Garg, Charles Markello, Michael F Lin, et al. Variation graph toolkit improves read mapping by representing genetic variation in the reference. *Nature biotechnology*, 36(9):875–879, 2018.

MRS$^+$12. Trudy FC Mackay, Stephen Richards, Eric A Stone, Antonio Barbadilla, Julien F Ayroles, Dianhui Zhu, Sònia Casillas, Yi Han, Michael M Magwire, Julie M Cridland, et al. The drosophila melanogaster genetic reference panel. *Nature*, 482(7384):173–178, 2012.

MTF$^+$21. Quirin Manz, Olga Tsoy, Amit Fenn, Jan Baumbach, Uwe Völker, Markus List, and Tim Kacprowski. Asimulator: splice-aware rna-seq data simulation. *Bioinformatics*, 37(18):3008–3010, 2021.

SEN$^+$23. Jonas A Sibbesen, Jordan M Eizenga, Adam M Novak, Jouni Sirén, Xian Chang, Erik Garrison, and Benedict Paten. Haplotype-aware pantranscriptome analyses using spliced pangenome graphs. *Nature Methods*, pages 1–9, 2023.

SPL$^+$14. Shihao Shen, Juw Won Park, Zhi-xiang Lu, Lan Lin, Michael D Henry, Ying Nian Wu, Qing Zhou, and Yi Xing. rmats: robust and flexible detection of differential alternative splicing from replicate rna-seq data. *Proceedings of the National Academy of Sciences*, 111(51):E5593–E5601, 2014.

SWWB$^+$18. Timothy Sterne-Weiler, Robert J. Weatheritt, Andrew J. Best, Kevin C.H. Ha, and Benjamin J. Blencowe. Efficient and accurate quantitative profiling of alternative splicing patterns of any complexity on a laptop. *Molecular Cell*, 72(1):187–200.e6, 2018.

TEH$^+$18. Juan L Trincado, Juan C Entizne, Gerald Hysenaj, Babita Singh, Miha Skalic, David J Elliott, and Eduardo Eyras. Suppa2: fast, accurate, and uncertainty-aware differential splicing analysis across multiple conditions. *Genome biology*, 19:1–11, 2018.

TGS$^+$19. Jim Thurmond, Joshua L Goodman, Victor B Strelets, Helen Attrill, L Sian Gramates, Steven J Marygold, Beverley B Matthews, Gillian Millburn, Giulia Antonazzo, Vitor Trovisco, et al. Flybase 2.0: the next generation. *Nucleic acids research*, 47(D1):D759–D765, 2019.

Supplementary Figures and Tables

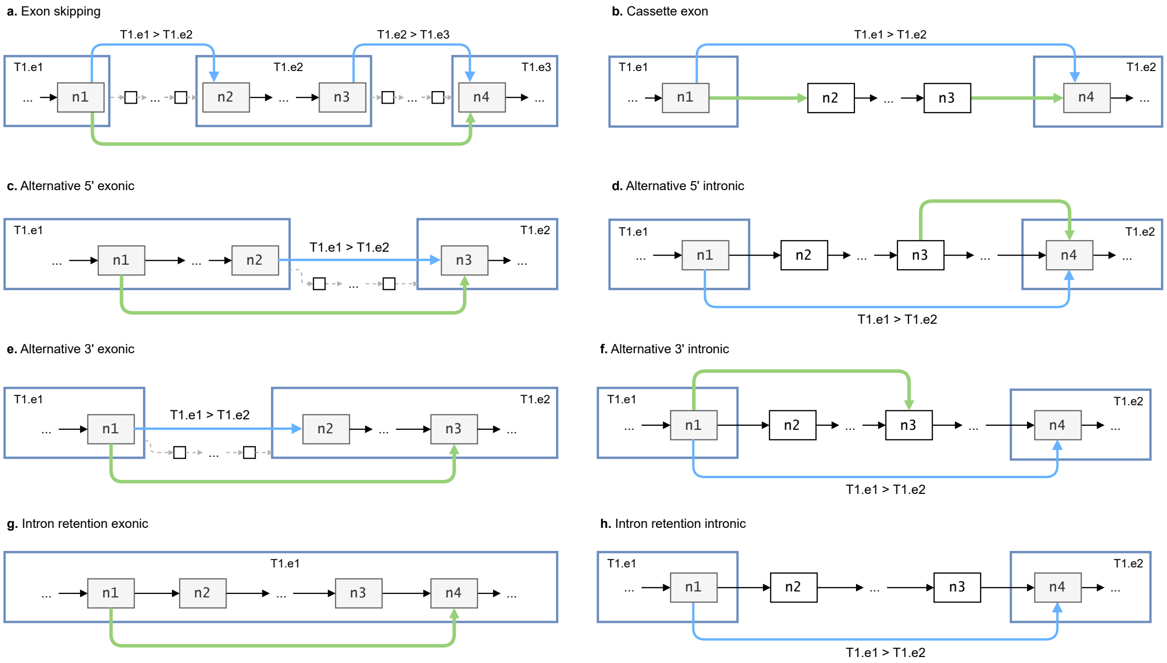

Fig. A: All the novel events expressed within the annotated spliced pangenome showing the different tags used. Haplotype information and weights are omitted for readability. Blue squares represent exons with their tags, blue edges are the annotated junctions with their tags, green edges are the novel links, grey vertices are exonic and white are intronic,

True Support ($\mathcal{W}$)	Exon Skippings	Alternative 3' Sites	Alternative 5' Sites	Intron Retentions
1	214	219	209	231
3	152	170	155	178
5	129	140	130	153
10	95	108	95	120
20	65	65	55	75

Table A: Number of events reported by `asimulator` and supported by at least $\mathcal{W}$ reads.

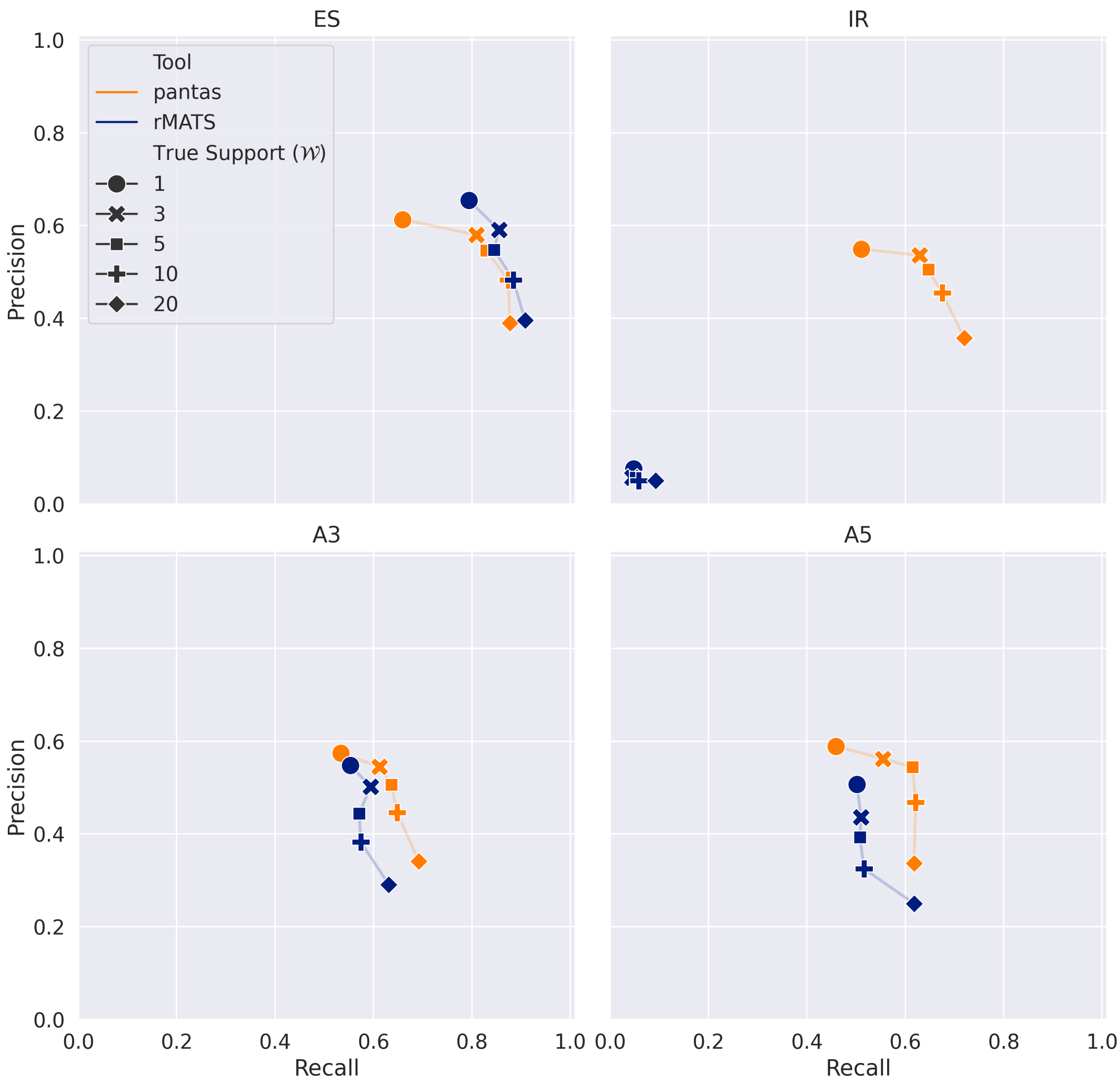

Fig. B: Results on simulated data from Drosophila Melanogaster (novel events). Results are broken down by event type (ES: Exon Skipping, IR: Intron Retention, A3: Alternative 3', A5: Alternative 5').

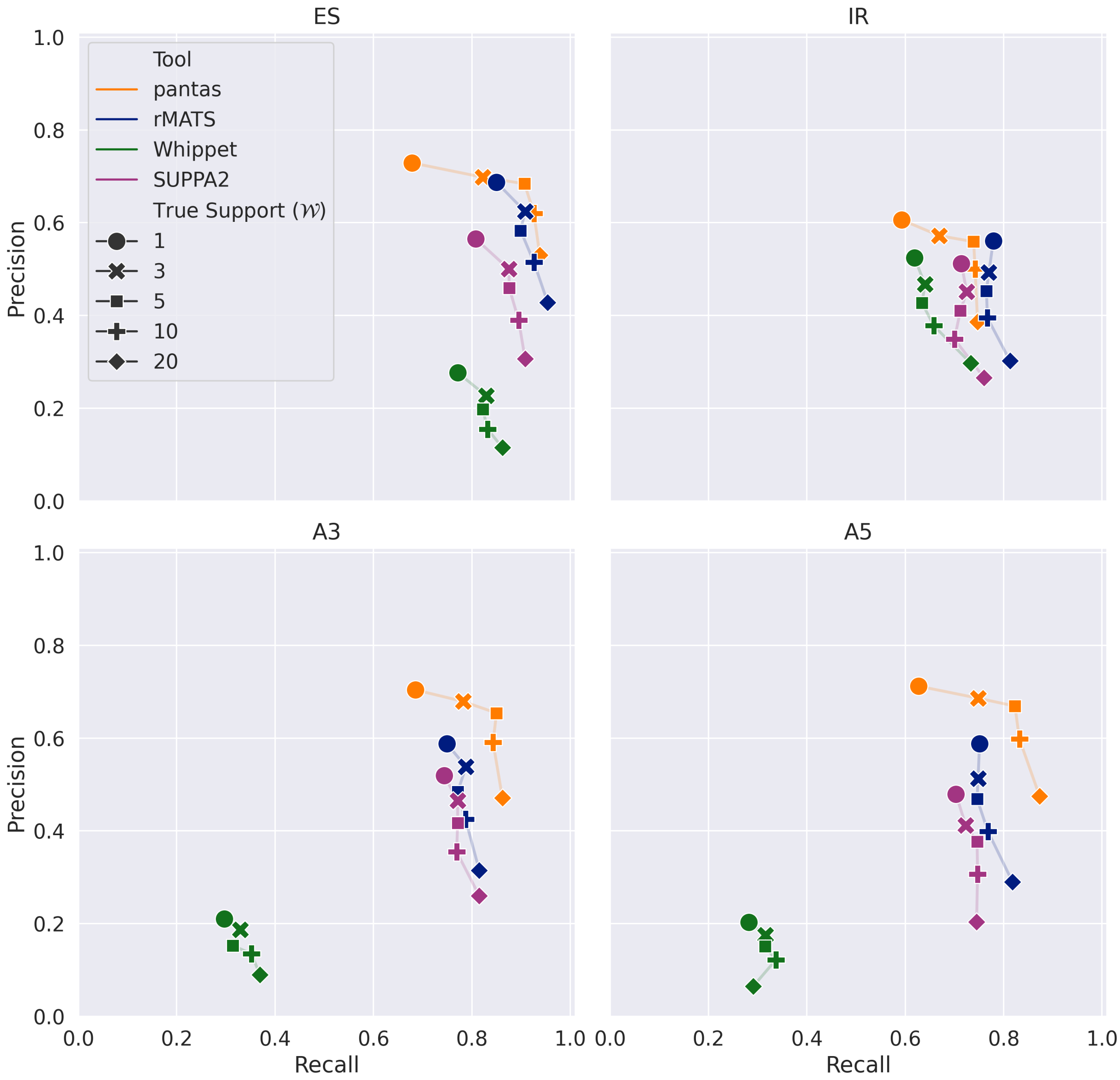

Fig. C: Results on simulated data from Drosophila Melanogaster (annotated events). These results are obtained running `pantas` setting its w parameter to 5 (minimum number of alignments needed to report an event). Results are broken down by event type (ES: Exon Skipping, IR: Intron Retention, A3: Alternative 3', A5: Alternative 5').

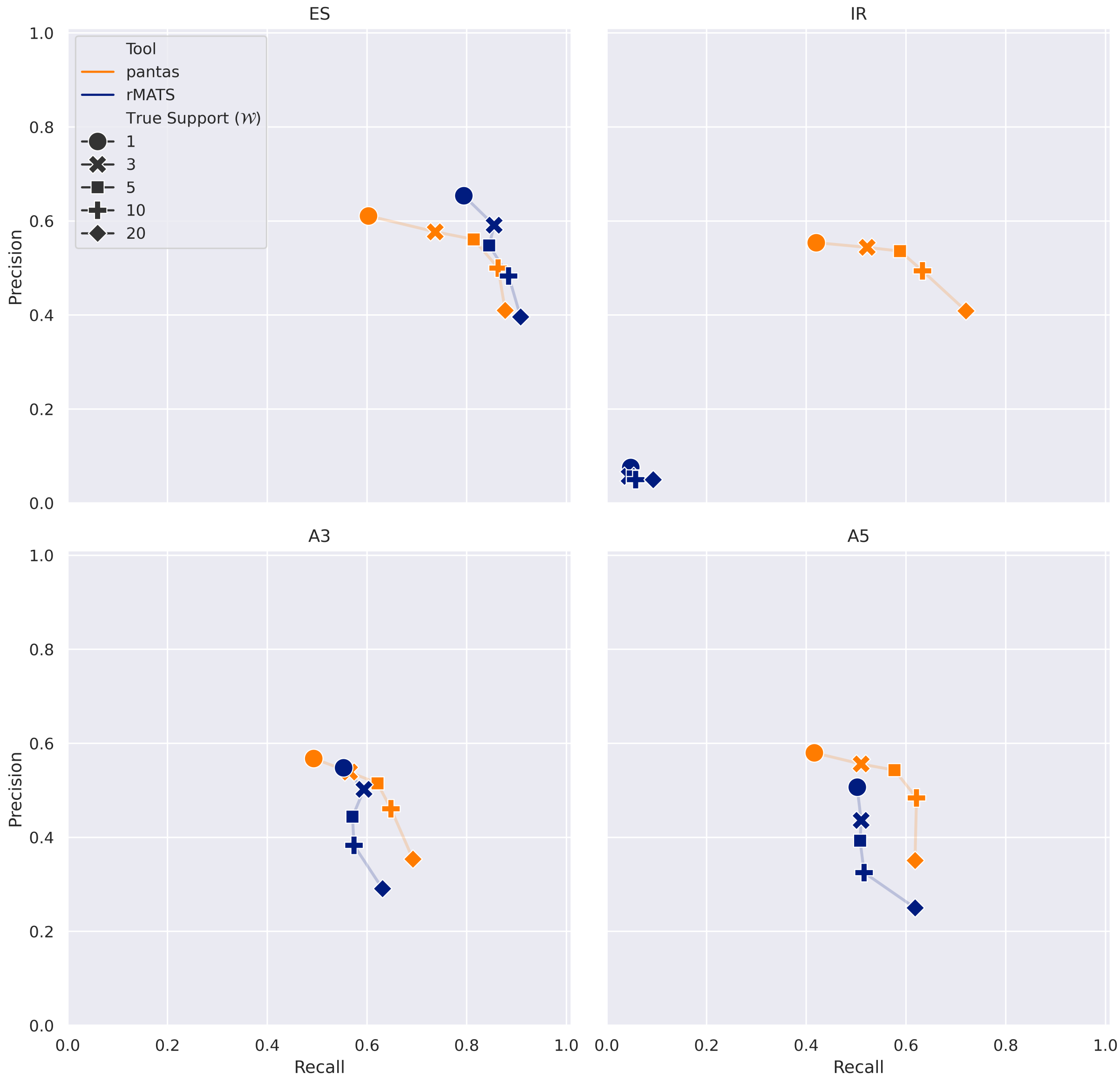

Fig. D: Results on simulated data from Drosophila Melanogaster (novel events). These results are obtained running `pantas` setting its w parameter to 5 (minimum number of alignments needed to report an event). Results are broken down by event type (ES: Exon Skipping, IR: Intron Retention, A3: Alternative 3', A5: Alternative 5').

$\mathcal{W}$	Event	Tool	TP	FN	FP	Prec	Rec	F1
1	ES	pantas	162	52	60	0.73	0.757	0.743
1	ES	rMATS	182	32	83	0.687	0.85	0.76
1	ES	whippet	165	49	430	0.277	0.771	0.408
1	ES	SUPPA2	173	41	133	0.565	0.808	0.665
1	IR	pantas	150	81	100	0.6	0.649	0.624
1	IR	rMATS	180	51	141	0.561	0.779	0.652
1	IR	whippet	143	88	130	0.524	0.619	0.567
1	IR	SUPPA2	165	66	157	0.512	0.714	0.597
1	A3	pantas	164	55	66	0.713	0.749	0.731
1	A3	rMATS	164	55	115	0.588	0.749	0.659
1	A3	whippet	65	154	244	0.21	0.297	0.246
1	A3	SUPPA2	163	56	151	0.519	0.744	0.612
1	A5	pantas	140	69	58	0.707	0.67	0.688
1	A5	rMATS	157	52	110	0.588	0.751	0.66
1	A5	whippet	59	150	231	0.203	0.282	0.236
1	A5	SUPPA2	147	62	160	0.479	0.703	0.57
3	ES	pantas	138	14	60	0.697	0.908	0.789
3	ES	rMATS	138	14	83	0.624	0.908	0.74
3	ES	whippet	126	26	430	0.227	0.829	0.356
3	ES	SUPPA2	133	19	133	0.5	0.875	0.636
3	IR	pantas	129	49	100	0.563	0.725	0.634
3	IR	rMATS	137	41	141	0.493	0.77	0.601
3	IR	whippet	114	64	130	0.467	0.64	0.54
3	IR	SUPPA2	129	49	157	0.451	0.725	0.556
3	A3	pantas	145	25	66	0.687	0.853	0.761
3	A3	rMATS	134	36	115	0.538	0.788	0.64
3	A3	whippet	56	114	244	0.187	0.329	0.238
3	A3	SUPPA2	131	39	151	0.465	0.771	0.58
3	A5	pantas	124	31	58	0.681	0.8	0.736
3	A5	rMATS	116	39	110	0.513	0.748	0.609
3	A5	whippet	49	106	231	0.175	0.316	0.225
3	A5	SUPPA2	112	43	160	0.412	0.723	0.525
5	ES	pantas	117	12	60	0.661	0.907	0.765
5	ES	rMATS	116	13	83	0.583	0.899	0.707
5	ES	whippet	106	23	430	0.198	0.822	0.319
5	ES	SUPPA2	113	16	133	0.459	0.876	0.603
5	IR	pantas	110	43	100	0.524	0.719	0.606
5	IR	rMATS	117	36	141	0.453	0.765	0.569
5	IR	whippet	97	56	130	0.427	0.634	0.511
5	IR	SUPPA2	109	44	157	0.41	0.712	0.52
5	A3	pantas	119	21	66	0.643	0.85	0.732
5	A3	rMATS	108	32	115	0.484	0.771	0.595
5	A3	whippet	44	96	244	0.153	0.314	0.206
5	A3	SUPPA2	108	32	151	0.417	0.771	0.541
5	A5	pantas	107	23	58	0.648	0.823	0.725
5	A5	rMATS	97	33	110	0.469	0.746	0.576
5	A5	whippet	41	89	231	0.151	0.315	0.204
5	A5	SUPPA2	97	33	160	0.377	0.746	0.501

$\mathcal{W}$	Event	Tool	TP	FN	FP	Prec	Rec	F1
10	ES	pantas	88	7	60	0.595	0.926	0.724
10	ES	rMATS	88	7	83	0.515	0.926	0.662
10	ES	whippet	79	16	430	0.155	0.832	0.262
10	ES	SUPPA2	85	10	133	0.39	0.895	0.543
10	IR	pantas	87	33	100	0.465	0.725	0.567
10	IR	rMATS	92	28	141	0.395	0.767	0.521
10	IR	whippet	79	41	130	0.378	0.658	0.48
10	IR	SUPPA2	84	36	157	0.349	0.7	0.465
10	A3	pantas	91	17	66	0.58	0.843	0.687
10	A3	rMATS	85	23	115	0.425	0.787	0.552
10	A3	whippet	38	70	244	0.135	0.352	0.195
10	A3	SUPPA2	83	25	151	0.355	0.769	0.485
10	A5	pantas	79	16	58	0.577	0.832	0.681
10	A5	rMATS	73	22	110	0.399	0.768	0.525
10	A5	whippet	32	63	231	0.122	0.337	0.179
10	A5	SUPPA2	71	24	160	0.307	0.747	0.436
20	ES	pantas	61	4	60	0.504	0.938	0.656
20	ES	rMATS	62	3	83	0.428	0.954	0.59
20	ES	whippet	56	9	430	0.115	0.862	0.203
20	ES	SUPPA2	59	6	133	0.307	0.908	0.459
20	IR	pantas	57	18	100	0.363	0.76	0.491
20	IR	rMATS	61	14	141	0.302	0.813	0.44
20	IR	whippet	55	20	130	0.297	0.733	0.423
20	IR	SUPPA2	57	18	157	0.266	0.76	0.394
20	A3	pantas	56	9	66	0.459	0.862	0.599
20	A3	rMATS	53	12	115	0.315	0.815	0.455
20	A3	whippet	24	41	244	0.09	0.369	0.144
20	A3	SUPPA2	53	12	151	0.26	0.815	0.394
20	A5	pantas	48	7	58	0.453	0.873	0.596
20	A5	rMATS	45	10	110	0.29	0.818	0.429
20	A5	whippet	16	39	231	0.065	0.291	0.106
20	A5	SUPPA2	41	14	160	0.204	0.745	0.32

Table B: Full results on simulated data from Drosophila Melanogaster (annotated events). Precision, Recall, and F1 are reported for all true support threshold tested ($\mathcal{W}$).

$\mathcal{W}$	**Event**	**Tool**	**TP**	**FN**	**FP**	**Prec**	**Rec**	**F1**
1	ES	pantas	141	73	89	0.613	0.659	0.635
1	ES	rMATS	170	44	90	0.654	0.794	0.717
1	IR	pantas	118	113	97	0.549	0.511	0.529
1	IR	rMATS	11	220	133	0.076	0.048	0.059
1	A3	pantas	117	102	87	0.574	0.534	0.553
1	A3	rMATS	121	98	100	0.548	0.553	0.55
1	A5	pantas	96	113	67	0.589	0.459	0.516
1	A5	rMATS	105	104	102	0.507	0.502	0.505
3	ES	pantas	123	29	89	0.58	0.809	0.676
3	ES	rMATS	130	22	90	0.591	0.855	0.699
3	IR	pantas	112	66	97	0.536	0.629	0.579
3	IR	rMATS	8	170	133	0.057	0.045	0.05
3	A3	pantas	104	66	87	0.545	0.612	0.576
3	A3	rMATS	101	69	100	0.502	0.594	0.544
3	A5	pantas	86	69	67	0.562	0.555	0.558
3	A5	rMATS	79	76	102	0.436	0.51	0.47
5	ES	pantas	107	22	89	0.546	0.829	0.658
5	ES	rMATS	109	20	90	0.548	0.845	0.665
5	IR	pantas	99	54	97	0.505	0.647	0.567
5	IR	rMATS	8	145	133	0.057	0.052	0.054
5	A3	pantas	89	51	87	0.506	0.636	0.563
5	A3	rMATS	80	60	100	0.444	0.571	0.5
5	A5	pantas	80	50	67	0.544	0.615	0.578
5	A5	rMATS	66	64	102	0.393	0.508	0.443
10	ES	pantas	83	12	89	0.483	0.874	0.622
10	ES	rMATS	84	11	90	0.483	0.884	0.625
10	IR	pantas	81	39	97	0.455	0.675	0.544
10	IR	rMATS	7	113	133	0.05	0.058	0.054
10	A3	pantas	70	38	87	0.446	0.648	0.528
10	A3	rMATS	62	46	100	0.383	0.574	0.459
10	A5	pantas	59	36	67	0.468	0.621	0.534
10	A5	rMATS	49	46	102	0.325	0.516	0.398
20	ES	pantas	57	8	89	0.39	0.877	0.54
20	ES	rMATS	59	6	90	0.396	0.908	0.551
20	IR	pantas	54	21	97	0.358	0.72	0.478
20	IR	rMATS	7	68	133	0.05	0.093	0.065
20	A3	pantas	45	20	87	0.341	0.692	0.457
20	A3	rMATS	41	24	100	0.291	0.631	0.398
20	A5	pantas	34	21	67	0.337	0.618	0.436
20	A5	rMATS	34	21	102	0.25	0.618	0.356

Table C: Full results on simulated data from Drosophila Melanogaster (novel events). Precision, Recall, and F1 are reported for all true support threshold tested ($\mathcal{W}$).

**Event**	pantas	rMATS	whippet	SUPPA2
ES	370	366	3205	140
A3	337	200	401	129
A5	367	189	385	131
IR	322	177	331	113
All	1396	932	4322	513

Table D: Alternative splicing events count for Drosophila Melanogaster experiments on real data.

**Event**	`pantas`	`rMATS`	`whippet`	`SUPPA2`
ES	553	1220	12030	755
A3	627	754	731	944
A5	630	638	705	909
IR	506	536	537	663
All	2316	3148	14003	3271

Table E: AS events count for Drosophila Melanogaster experiments without any filtering on minimum $\Delta\psi$, minimum probability, and p-value.

Tool	Time (min.)	RAM (GB)
vg index	128	20
vg mpmap	388*	3
pantas weight	26*	5
pantas call	11*	16
pantas quant	1	1
STAR index	1	4
STAR align	20*	6
rMATS	2	1
salmon index	0.5	1
salmon quant	1*	1
SUPPA2	0.5	1
whippet index	2	1
whippet quant	9*	1
whippet delta	1	1

* average over the 6 samples.

Table F: Efficiency results on real data from Drosophila Melanogaster. Time usage is reported in minutes. Peak RAM usage is reported in GB.

Tool	Time (min)	RAM (GB)
`shark`	2*	2
`vg index`	2	8
`vg mpmap`	20*	3
`pantas weight`	1*	1
`pantas call`	1*	1
`pantas quant`	1	1
STAR index	43	38
STAR align	9*	32
rMATS	9	2
salmon index	2	1
salmon quant	2*	3
SUPPA2	13	1
whippet index	45	5
whippet quant	29*	6
whippet delta	2	1

* average over the 6 samples.

Table G: Efficiency results on real Human data. Time usage is reported in minutes. Peak RAM usage is reported in GB.

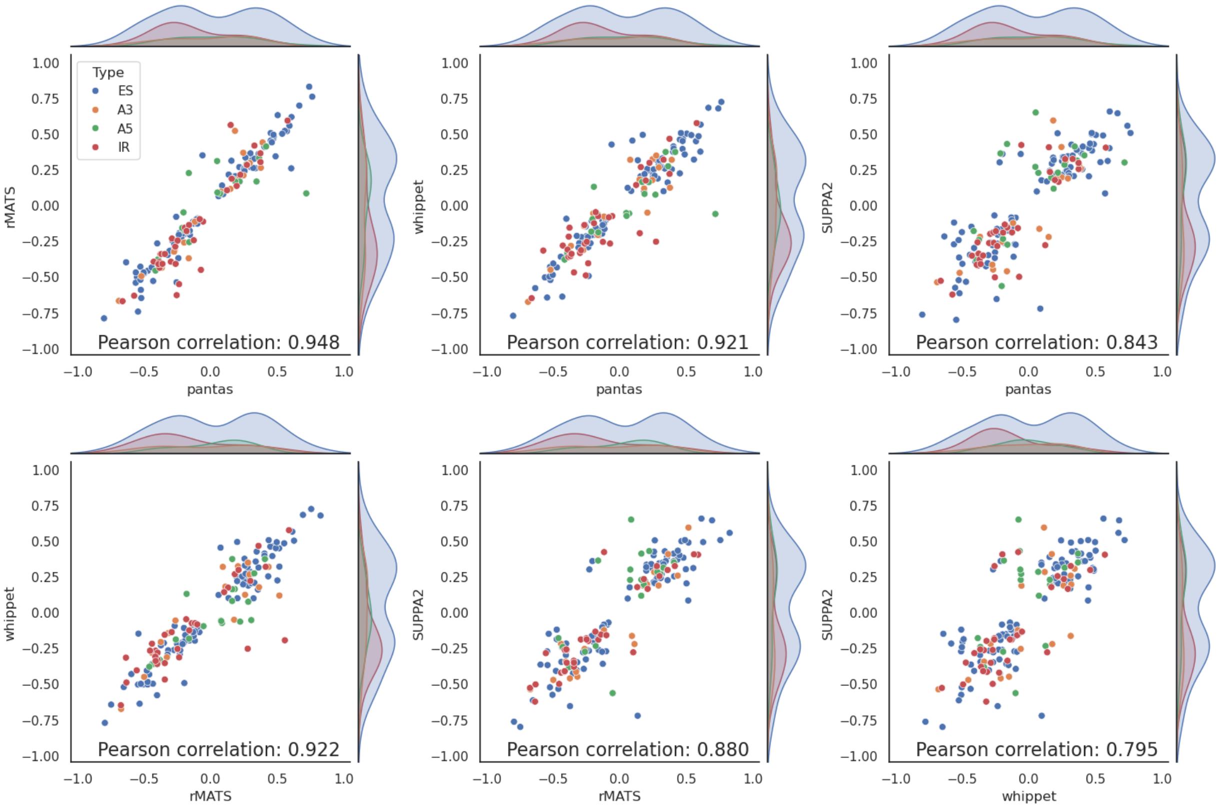

Fig. E: Results on real data from Drosophila Melanogaster. All-vs-all correlation plots of the $\Delta\psi$ reported by the considered tools for the 164 events shared among the 4 tools. Each point is an event color coded based on its type.

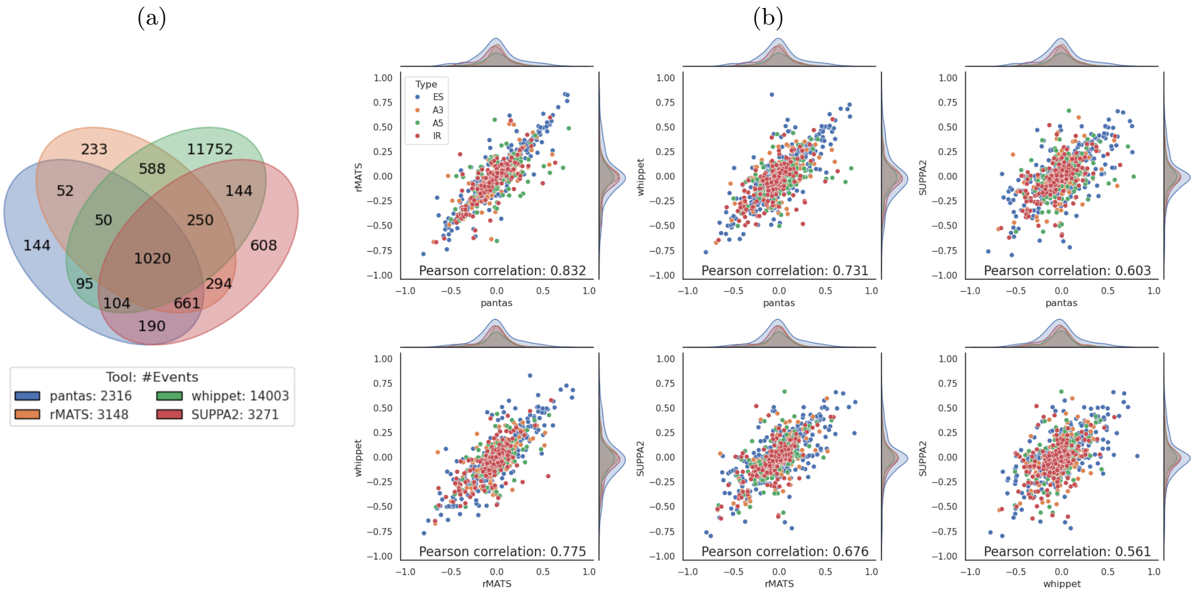

Fig. F: Results on real data from Drosophila Melanogaster (all events). (a) Venn diagram showing the number of AS events reported by each tool. (b) All-vs-all correlation plots of the $\Delta\psi$ reported by the considered tools for the 1020 events shared among the 4 tools. Each point is an event color coded based on its type.

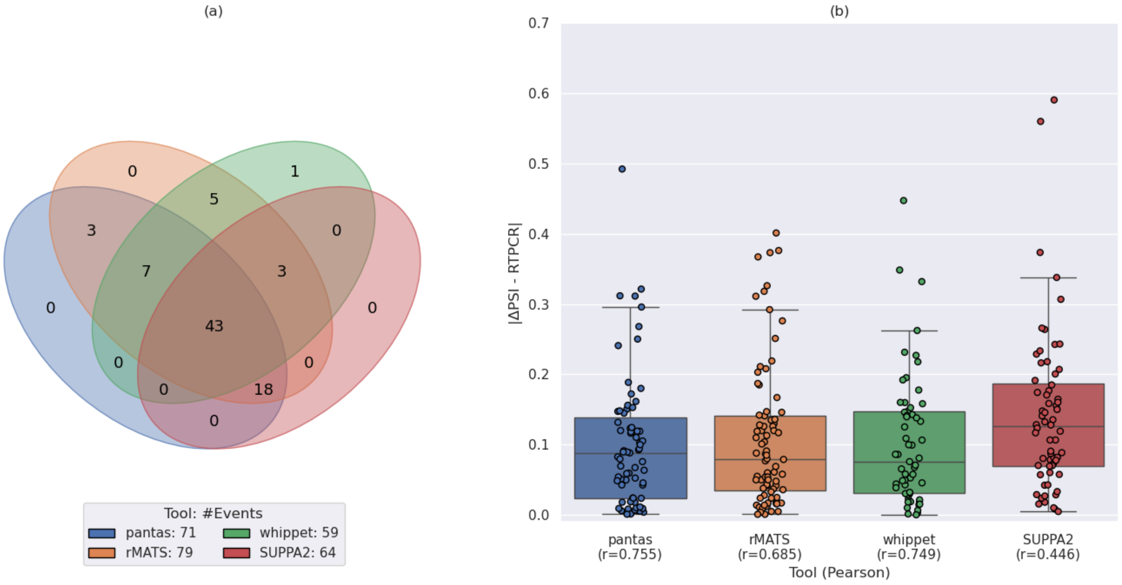

Fig. G: (a) Venn diagram showing the number of AS events reported by each tool. The legend reports the total number of events reported by the tool. (b) Boxplot showing the distribution of the difference between the $\Delta\psi$ predicted by each tool and the $\Delta\psi$ provided by RT-PCR. The x axis labels report the tool name and the Pearson correlation (r) between the predicted $\Delta\psi$ and the RT-PCR $\Delta\psi$.

.
