# Supplementary material for: Differential quantification of alternative splicing events on spliced pangenome graphs
Source: PLoS Comput Biol. 2024 Dec 9;20(12):e1012665. doi: 10.1371/journal.pcbi.1012665 (PMC11658704; doi:10.1371/journal.pcbi.1012665)
Supplement: Attachment — Submitted filename: pantas-response.pdf [file pcbi.1012665.s002.pdf]

Responses to Reviewers

"Differential quantification of alternative splicing events on spliced pangenome graphs"

We would like to thank the anonymous reviewers for their detailed comments. In our opinion our revision addresses all reviewers concerns. Our responses to their comments are detailed below.
This is a summary of the main changes:

- We completely revised our model to fully support haplotype-aware transcripts. The model is not reference-centric anymore and it is now possible to detect events involving transcripts that do not lay on the reference transcripts. Anyway, the implementation still needs a reference haplotype, since it is mainly based on the `vg` toolkit: this reference is used as the backbone while building the graph, but then `pantas`detection is reference-agnostic (if not in its last step, where events are remapped back to the reference haplotype).
- This change required us to reimplement most of the core parts of `pantas`. We revised our methodology to construct and annotate a spliced pangenome. While doing so, we realized that it was more convenient (and clearer) to describe a single construction methodology. There is no more a separate "gene-centric" construction but its description has been included in the main construction. While building a spliced pangenome, `pantas` allows to simplify it (before indexing) using one of three different methodologies (described in the corresponding section).
- We entirely reran all the experiments and we updated the results accordingly.
- Regarding simulated data, we decided to present the results in a different way. Instead of providing a single value of precision/recall/F1, we analyzed how these accuracy values are affected by the true coverage of the events (reported by the simulator). Moreover, we would like to note that while performing the new analysis, we realized that we were filtering the truth sets wrongly. This error lead to an increase of accuracy for all tools. We have now fixed it.

Editor's comments

Editor: Please pay attention to the comments on data, code and reproducibility. Per PLoS data policy the code for reproducing and replicating the analysis must be provided.

We respectfully point out that the "Experiments" section of the README in the github repository of pantas briefly described how to replicate the experimental evaluation described in the paper (`https://github.com/algolab/pantas?tab=readme-ov-file#experiments`). All `pantas` code is freely available on the github repository and all data we used is publicly available. The sub-directories of the "exps" directory contain additional instructions that can be followed to download the data and replicate the experiments. We have added a clear statement on how to perform replication.

> ***Code and data availability***
> *`pantas` is open source and publicly available at `https://github.com/algolab/pantas`. The repository also contains all the scripts used in the experimental evaluations described in the manuscript. All data is publicly available. Annotated splicing pangenome graphs are available at `https://zenodo.org/records/13740102`. Instructions on how to download the data, preprocess them, and run the analyses are available at `https://github.com/algolab/pantas?tab=readme-ov-file#experiments`.*

Reviewer #1

Reviewer: Ciccolella et al. describes the pandas tool to detect alternative splicing events using annotated spliced pangenomes. This is the first pangenomic method for AS detection. Even though the idea itself is interesting, the manuscript could be improved by providing more evidences and examples to demonstrate the advantage of this new method.

We thank the reviewer for the suggestions he gave and the doubts he raised. We heavily revised the manuscript and we hope that the new version addresses most of its concerns.

Reviewer: In section 2.1, what is the formal definition of a "walk"? Was the annotated spliced pangenome constructed by the authors, or readily available from existing resources? If it was constructed by the authors, is it made publicly available to the community?

We added the definition of walk in Section 2.1:

> *A* walk *in a graph is a sequence of vertices connected by edges.*

All data (references, annotations, variant databases, and real RNA-Seq datasets) are already publicly available. Instructions to fetch these data and to simulate RNA-Seq reads can be found in the pantas github repository (subdirectory in the "exps" directory). We uploaded the annotated spliced pangenome we created for our experimental evaluation on zenodo. We added a *"Code and Data availability"* section at the end of the main manuscript.

Reviewer: In section 2.2, what is the tolerance level to consider a splicing event novel? If a splice junction only differs from an annotated one by 1 bp, is it still considered novel?

There is no tolerance level to consider a splicing event novel. So, even a 1-bp shift of splice site will be considered a novel event. Anyway, we believe this is a quite extreme case that rarely occurs in practice - since aligner scoring models usually penalize indels more than substitutions (making a single substitution more convenient than an indel of size 1). Longer indels close to splice sites are not filtered out by `pantas` but can be easily filtered out by the user (in any case, we would like to note that, at least in our experimental evaluation, `pantas`'s precision is not affected by these short shifts since it is quite uncommon that many reads share the same indel at the same position — if due to sequencing errors).

Reviewer: After reading the whole manuscript, it is not clear what the exact output of pantas look like. Can the authors provide a more specific description with examples?

The output of pantas is a text file (CSV) describing all events detected by pantas. The description of this format and what each column represents is available in the README of the github repository (`https://github.com/algolab/pantas?tab=readme-ov-file#custom-output-format`). In our opinion, these implementation-level details should not be in the manuscript.

Reviewer: Is pandas only designed for short reads? Can it be used with long reads?

We would like to note that, in our opinion, `pantas` model does not depend on sequencing technology. However, the tool is currently limited to short-reads data. We made this clearer in the abstract.

Reviewer: The manuscript compares pantas with three existing tools and all three tools are able to evaluate statistical significance. I wonder why pantas does not perform statistical validation using similar methods as those used by other tools.

We think that this is a very interesting problem, but it is worth of a future direction. Given the complexity of the classification task, we focused on formalizing a definition of the problem and experiment one possible solution. These results can then be used as basis for a rigorous work on the quantification side of the event, once the classification is solved.

Reviewer: Why didn't the author compare pantas with leafcutter, which is also a popular tool?

We did not include leafcutter in our analysis since it does not natively support AS event classification. It rather provides a set of introns that need to be visually inspected to retrieve the type of event. Here some issues from the leafcutter github repository:

- `https://github.com/davidaknowles/leafcutter/issues/120`
- `https://github.com/davidaknowles/leafcutter/issues/110`
- `https://github.com/davidaknowles/leafcutter/issues/235`

Reviewer: The current simulation is based on Drosophila genome. What if the simulation is done using the human genome, which may have more complex splicing events? Additionally, can the authors use simulation to evaluate how method performance is impacted by sequencing depth?

Since asimulator simulates a RNA-Seq dataset while introducing AS events (using "fixed rules"), the choice of the organism does not affect the set of true AS events: asimulator creates a template transcript per gene and then it alters this transcript introducing AS events and creating alternative transcripts (this is described in more details in the Supplementary Material of the manuscript).
We decided to perform a simulation on an organism with a genome not too small nor as big as human genome. We opted for the Drosophila to keep the simulation as efficient as possible without removing most of the complexity of AS detection, since Drosophila has a quite comprehensive gene annotation.
Regarding the last point on sequencing depth, we did not perform a coverage titration analysis but we updated the results presentation in a way that, we believe, may shed more light on the impact of sequencing depth. Indeed now, instead of presenting a single value (P/R/F1) per tool, we analyzed how the accuracy of each tool change while varying the true coverage of the AS events simulated with asimulator. `pantas` provides a parameter to set the minimum

junction support to call an event (by default set to 3). The new results show that `pantas` detection depends on this parameter but when the true coverage of an event is higher than the chosen value, `pantas` did manage to achieve greater accuracy than other tools. Those parts can be found in the updated Section 3.1 of the manuscript.

> **Reviewer:** "As done in our previous experiment, we ran all tools with their default parameters, except for pantas, for which we set the minimum junction support to 5, in order to achieve a good trade-off between true AS events and false calls." This approach leads to the question that how users could select this parameter in their own analysis to achieve a good tradeoff?

The support threshold is used to offset possible changes in coverage. However, in RNA-Seq samples the coverage is not always uniform since it mainly depends on transcript expression levels. The default value of this parameter is now set to 3 and this value is used in all our experiments.
However, we are not able to suggest a particular value to be used in all cases, since different scenarios may require different values for the parameter. For example if one is interested in rare AS events (i.e., events with low expression levels), they may prefer really low (or even zero) minimum support. On the other hand if one is interested in only the two most expressed transcripts they would prefer to have this value set close to 40 or 50% of the expected coverage. For this reason, we allow the users to set this parameter to suit their specific needs.
We added this paragraph (at the end of Section 3.1):

> *Even though the accuracy of* `pantas` *heavily depends on the chosen value of its w parameter, we believe that these results show that, when properly set, it enables accurate AS event detection. To test this claim, we reran all the analyses increasing the w parameter to 5 (results for annotated and novel events reported in Supp.Figures S3 and S4, respectively). As expected, this time* `pantas` *managed to achieve the best accuracy when the true event support (ω) is ≥ 5. However, setting this parameter is not easy since RNA-Seq coverage is not always uniform. Anyway, we believe that the default value (3) should be used in most of the scenarios. Future efforts will be focused in removing this dependency on the w parameter by including a statistical validation of* `pantas` *results.*

> **Reviewer:** "An extensive experimental evaluation shows that pantas achieves results that are comparable with those of state-of-the-art methods based on a linear reference". Can the authors clarify when it is advantageous to use pantas other than state-of-the-art methods?

zThe work of *Sibbesen et al.* has shown a clear advantages in the use of a haplotype-aware pangenome graph in the analysis of transcripts. However, their work leaves open the problem of how to perform tasks such as detection of AS events and AS differential quantification in pangenome graphs. The main goal of `pantas` is to fill this gap in order to take advantage of variants in transcripts while performing these tasks. Current work shows that a haplotype-aware AS

quantification is feasible using pangenome graphs and it achieves competitive if not better results than those of state-of-the-art methods based on a linear reference. Since `pantas` includes a representation of variants in transcripts, it allows a downstream analysis of AS events that may be related to specific alleles, thus bringing potential improvements in the future in the use of a pangenome in AS detection. In the revised work, we focused on the detection of AS events from haplotype-aware annotations. We believe that the post-processing of `pantas` output (i.e., the AS events) can lead to interesting discoveries but it will need additional investigation and a more thorough analysis of real data. In our opinion, this direction, although compelling, goes out the scope of our paper.

Reviewer: The manuscript describes how to prepare input files for pantas in supplementary sections. It will be helpful to incorporate these steps in the github tutorial with example datasets.

Instructions on how to prepare the input for pantas are now available in the README of the github repository. The construction of the annotated spliced pangenome is provided as a snakemake workflow and can be simply run via a single command (as described in the README). Example data are provided and instructions on how to use them can be found in the "Example" section of the README (`https://github.com/algolab/pantas?tab=readme-ov-file#example`).

Reviewer #2

Reviewer: This paper presents a new tool, pantas, which is capable of detecting alternative splicing using RNAseq reads mapped to a spliced pangenome graph, supporting standard formats for both. The pantas tool is compared to other tools for detecting alternative splicing, with results suggesting that the tool does in fact work to detect alternative splicing. The manuscript also includes some formal definitions for alternative splicing event types in a graph context, and in the supplement there is a description of a method for building a smaller spliced pangenome graph suitable for analyzing alternative splicing in only a small panel of genes, with reduced runtime costs. As presented, the pantas tool seems competitive in accuracy, but the experiments presented do not actually test whether its biggest purported feature, the ability to leverage pangenome information, is actually responsible for its performance. I also have some concerns about how the paper communicates what pantas does and the core ideas that make it work, that I would like to see resolved before it is published.

We completely revised the `pantas` model and we heavily updated the manuscript in order to address the main concerns of the reviewer.

Reviewer: In terms of experiments, while the authors' experiments demonstrate that pantas is a competitive alternative splicing detection tool, the paper attributes its effectiveness to its use of pangenome information without actually testing that hypothesis. It could be that pantas is just a better tool than the others, and would perform just as well even if it were given no information on variation in the population, and run on a graph that was merely a splicing graph. The authors should include a negative control that shows how well pantas performs without access to pangenome information, if they want to argue that the pangenome information is actually responsible for pantas's accuracy.
They also could consider doing an analysis to somehow directly address reference bias and whether pantas is actually benefiting from its reduction. It has been previously shown that mapping to a pangenome can reduce reference bias, but it is not clear how much a reduction in reference bias specifically helps pantas's performance, as opposed to other factors. (It might be that all benefit from using a pangenome in pantas can be safely assumed to be due to reduced reference bias, but the benefit of having pangenome information is, as mentioned above, not directly measured.) If the authors don't measure the effect of reference bias reduction, they shouldn't write as if they know that it mediates a positive effect on pantas' performance.

We thank the reviewer for raising this issue. Based on his comments, we have revised the manuscript to avoid any reference to reference bias reduction. As the reviewer stated, our goal was to provide a first approach for AS event quantification from spliced pangenome that was competitive w.r.t. state-of-the-art in order to show that pangenome can work in this context as well. We believe that the new version, which is reference-agnostic and can work with haplotype-aware transcripts, should help in better convey this message (we updated the manuscript accordingly). Regarding the reference bias reduction, we were grounding our statements on some sort of transitivity-based conjecture (as the reviewer pointed out): since the usage of spliced pangenome proved this reduction and `pantas` is built on an "extended" spliced pangenome (very similar to that used in *Sibbesen et al.*), then `pantas` itself benefits from the reduction. We agree that this was too hastily written and we rephrased all sentences referring to the bias reduction.

Reviewer: In terms of communication, I found the paper somewhat difficult to understand because I had an idea of what a pangenome-aware alternative splicing detector should do, and pantas doesn't do that. It uses a spliced pangenome graph, but instead of accounting for annotated transcripts that freely take paths through the graph, it uses a set of transcripts T that are required to be defined *only on the linear reference*, and it postulates a set of haplotypes H which it does not appear to use. Despite being told that any edges in transcripts that aren't in the reference walk R are necessarily splice edges, and that any vertex on a transcript walk "represents an exonic region of the reference genome", and despite being told and shown in Figure 1 that transcripts exist on the linear reference and both variants and introns are always outside of them, I got midway through writing this review before I figured out that, as currently implemented, pantas can't deal with known transcripts that leave the linear reference. Depending on how the authors define "exonic" vertices, which doesn't seem to be formalized, pantas as described may or may not be capable of discovering novel alternative splicing events that could not ever be added to its input database as part of any transcripts, because the junctions connect to non-reference vertices. What I wishfully thought I was looking at through the whole paper and well into writing the review was a tool that in fact made some attempt to deal with the interactions between the concept of alternative splicing and the concept of genomic variation in gene structure. Part of this may be attributable to my poor reading skills, but I think part of the reason for the misunderstanding is that the authors, when speaking less formally, write things like how they have "a formal definition of Alternative Splicing (AS) events on a graph structure" and that their work "paves the way to the adoption of spliced pangenomes for pantranscriptomic analysis, where genes are annotated w.r.t. a pangenome and not a single reference genome". They do indeed have *a* definition of alternative splicing events on a graph structure, but it is a definition that excludes most of what I think is interesting about the thing being defined, and which operates almost exclusively on an embedded linear reference path. And while what they describe as "spliced pangenomes for pantranscriptomic analysis" is more or less exactly what I wanted the authors to be doing, the work presented here doesn't "pave the way to the adoption" of it because it doesn't actually do it at all, or even provide a theoretical framework that would encompass it. There's a point in the conclusion about how pantas "can also work with haplotype-aware gene annotations" that go off the linear-reference, but if this is actually already true of the implementation then the theoretical model presented doesn't describe what pantas actually does.

To fix this, I think the authors first need to revise their abstract and introduction to communicate the message that, instead of a full treatment of alternative splicing on graphs, they actually have a basically-linear approach to alternative splicing. But, because it happens to operate on a linear path embedded in a pangenome graph, it can leverage pangenome technology to get and then directly use better read mappings, without any sort of mapping surjection step. Possibly it can *detect* novel alternative splicing events that create transcripts not representable against the linear reference. If so, this would support the things the authors want to say about how their tool is a kind of stepping-stone or jumping-off point that helps the field move towards a full treatment of alternative splicing on graphs, because it would enable researchers to start compiling a database of alternative splicing events observed to involve material outside the linear reference.

We completely revised our model and we re-implemented `pantas` to natively support haplotype-aware transcripts. Now transcripts can lay on any haplotype. Indeed, we also removed the reference from our model, although it is still needed in the implementation to project the transcript from the gene annotation (referring to the reference genome) to the haplotypes - as suggested at `https://github.com/vgteam/vg/wiki/Transcriptomic-analyses#pantranscriptomes`. We also

updated the output of `pantas` to take into account haplotype-aware transcripts: now `pantas` reports all haplotype-aware transcripts involved in an event. Moreover, as a technical note (described in the README of the repository), when all the junctions of an event come from a transcript (or multiple transcripts) that do not lie on the reference path, it is tagged as "haplotype" events so that the user explicitly know what is going on. We hope that the revised model and tool fix the issues raised by the reviewer.

> **Reviewer:** The authors also also need to make it somehow more clear that while they use the spliced pangenome graphs from their reference 33, they have decided not to use "pantranscriptome" set of haplotype-specific transcripts from that same work, or the approach to generalizing linear-reference gene annotations to graphs that the "pantranscriptome" concept relies on, or even the haplotype set H they invoke in their definitions. They might want to provide some justification for not using these, or at least for not allowing a pantranscriptome to be plugged in as the transcript set. It would seem to provide a way to at least get SNPs and small indels into the transcripts, thus allowing for things like a splice site that may or may not actually exist in a haplotype, and it is supposedly an output of the vg rna tool that pantas already relies on. If the authors just don't want to deal with the complexity of variation/alternative splicing interactions right now, they need to write a paragraph explicitly kicking that can down the road so that nobody can make the same mistake I did and assume they are dealing with it here and just forgot to explain how.

We refer the reviewer to the previous comment. We updated the model and the tool to use a pantranscriptome addressing the concern of the reviewer. We did not used the already available graphs from the *Sibbesen et al.* paper since we could not find a way to annotate those graphs using our definition of splice junction. Hence, we decided to wrap most of the steps needed to build a pantranscriptome (with additional steps required for annotating it) in a Snakemake pipeline that is available in the `pantas` github repository. In our opinion, this is a very convenient way to allow the creation of an annotated spliced pangenome for any organism.

> **Reviewer:** Leaving aside the treatment of variation, I also found the order in which the paper presents its treatment of read information confusing. In section 2.1, the authors begin with their formal model of a spliced pangenome graph, which has a set of haplotypes H and a set of transcripts T, on top of a variation graph with vertices and edges, and then extending this to an annotated spliced pangenome, where the vertices have sets of associated transcripts and exons, and the splice edges have sets of associated transcripts. They then launch into section 2.2 and spend two whole pages mathematically formalizing a taxonomy of alternative splicing events. But then it is not until section 2.3, nominally about pantas itself, that we get the key point that the graphs we have been talking about are supposed to have already been augmented, and have their vertices split wherever the sequencing reads propose new splice edges, and those splice edges added to the set of edges. This is necessary information for understanding the preceding sections, and needs to be introduced earlier. I would move some discussion of the concept of what I might call an "augmented annotated spliced pangenome graph", which is what is actually being operated on, to the section where the graphs are being defined, along with some kind of formalization of how read information is modeled (is it just coverage counts or are the read walks or base-level alignments used?) and the formal concept of "covered" for vertices used later (since w(e) seems to only be defined for splice edges).

We added an intuitive description of `pantas` at the beginning of Section 2. We hope this addition addresses the problem.
However, we decided to not change the order of the subsections since we believe that the formal description of the `pantas` model is one of the main contributions of our manuscript and the description of the `pantas` pipeline should build upon such formal description.

> **Reviewer:** I had trouble understanding the definitions of event types in section 2.2. The authors introduce the concept that some alternative splicing events are "annotated" and that some are "novel", but then they use this split as the first division in a taxonomy, going on to formally define some "annotated alternative splicing events" and some "novel alternative splicing events". This is driven mostly (it seems) by technical concerns about how to phrase the definitions without being allowed to refer to both options as members of the transcript set. There are then, for novel events, "intronic" and "exonic" flavors of almost all the event types, depending on which side of the alternative splicing event is the novel one. The most important thing about an alternative splicing event isn't usually thought to be whether it is in the database already or not (which is a non-biological artifact of what we've managed to put in the database). The authors don't do enough to warn the reader or justify why they are about to present this weird taxonomy, so I got dumped into a bunch of formal definitions without a clear idea of why they were structured and organized this way. I would revise the taxonomy to at the top level have the actual biological type of alternative splicing going on, and then within each give the three formal definitions for how it is detected when both sides are annotated, only the first alternative is annotated, and only the second alternative is annotated.
> Moreover, the formal definitions presented here require a lot of decoding to extract the information about what distinguishes different event types from each other. Why is there no such thing as an annotated "cassette exon"? If pantas tells me it has discovered an "intron retention exonic" alternative splicing event, is that the one where the transcript with the intron left in is the novel one, or the annotated one? I could probably follow the formal definitions through to their implied conclusions to work this out, but the paper would be much improved if it explained the intuitions behind the taxonomy and its chosen naming scheme in English, before delving into the set theory, because then the reader would understand the taxonomy and thus already expect to see the right things when reading each formal definition.

We thank the reviewer for the suggestion, but instead of mixing annotated and novel events as suggested, we have an updated classification which we think is easier to understand: we first focus on the easier class of annotated events and then we move to the much more complex class of novel events. Indeed, we believe that once the reader has a clear idea of what annotated events are defined, it is much easier for them to understand the novel ones.
We would like to note that this classification in annotated and novel (or de-novo) events has no biological motivation, as the reviewer points out, but has a strong computational motivation, as methods for detecting annotated events are completely different from those detecting novel events [1]. We updated the beginning of the section intuitively describing the two classes:

> *An AS event is said to be* annotated *if the junctions of the two transcripts involved in the event are already annotated, i.e., the edges representing the junctions are already present and annotated in the*

[1] `doi.org/10.1093/nargab/lqad044`

input graph. On the other hand, an event is said to be novel *if the splice junctions of only one of the two transcripts are already annotated and the junctions of the other transcript are supported by read alignments only.*

We understand that the formalization of all these different events might result tedious to go through, but we believe that the characterization of the problem is one of the contributions of this paper, especially regarding the translation of linear events to a graph structure.

Reviewer: I also think the analysis of the annotated event detection rates of the different tools (Table 1) needs to be better communicated, because having read it I'm confused about the notion of an annotated alternative splicing event existing or not existing in a particular read set. For novel alternative splicing events, a framework of detection makes sense because what the reads are doing is an alternative to what's in the annotation set. The annotation set might have all transcripts include a certain exon, but the reads attest to that exon being skipped some or all of the time, so you can detect that, as an alternative to what the annotations say, the exon is sometimes spliced out, which is a thing you didn't know from the annotations. For pantas specifically, the authors give definitions of when an alternative splicing event that is known to be possible from the annotations is to be considered "detected" by pantas: there needs to be read coverage of particular graph elements, generally those corresponding to one side of (i.e. one of the two classes of isoforms distinguished by) the event. These definitions are all given in the context of a single read set; pantas doesn't appear, for event detection purposes, to care about whether reads are present in one, the other, or both of two conditions being differentially compared. If pantas fails to detect an annotated alternative splicing event that was in the simulated data, this could be because the necessary read coverage was not there in the mapped reads. But how is it possible for it to ever call a false positive, unless the event definitions or transcripts used by pantas somehow systematically disagree with those coming out of the simulation tool? Is pantas's notion of annotated event detection (i.e. whether alternative splicing is possible and one alternative has good coverage) being compared against the simulator's idea of whether it put differentially-frequent alternative splicing into a pair of read sets? Because I don't think those would make sense to compare against each other.

We agree with the reviewer that the presentation of the simulated results was confusing. We then decided to completely overhaul it. Now we present the results varying the coverage reported by `asimulator`. We believe that this should communicate better what we mean by detection of annotated and novel events (since the accuracy computation primarily depends on how many reads support the events). We reply to the other concerns point-by-point:

1. The framework described by the reviewer in the novel settings is correct - with the addition of the dependency on simulated support
2. In the annotated scenario, the two isoforms are both in the annotation but the capability of the caller mainly depends on the correctness of its event definitions and the quality of its preprocessing (read alignment or transcript quantification).
3. We confirm that event detection is done on each replicate independently. Event quantification is done on the two conditions and differential quantification is done by comparing them.

4. We refer to points 1 and 2 to understand why there could be a false negative. We agree that false positives are a bit more confusing. We recall that we filtered out from the truth all events with $\Delta\psi < 0.05$. It may happen that a caller detected one of this events with greater $\Delta\psi$. Or it may happen that some reads have been wrongly aligned over a junction, making a tool wrongly believe that an event is supported by many reads (while, actually, it is supported by less reads - less than ω, that is the minimum support we used to create several truth sets in our new evaluation).

Reviewer: Also in Table 1, What is SUPPA2 doing to get perfect recall? What is pantas doing to get perfect precision and recall for A3 and A5 events specifically, and if it can genuinely always get them right 100% of the time, why can't it do the same for the other event types? Are the numbers of events such that it makes sense that a tool could get a perfect score by merely being very accurate, or are some tools exactly able to match the simulator's notions of annotated events because all the information needed is encoded in the transcript annotations it produces, which they have access to?

While performing the new analysis on simulated data, we realized that we were filtering the truth set in a wrong way. This error was improving the results of all tools. The new values (presented in Figure 3 and in the corresponding Supplementary Tables) are lower and no tool achieves 100% recall or precision. Moreover, we reported the number of events on each truth set we considered (depending on the minimum simulated support ω): this should give a better picture of the accuracy numbers presented in the manuscript.

Reviewer: Also, as a test of the event definitions in pantas, this analysis falls short because it considers only SNPs. It doesn't look at a graph with any particularly difficult graph structures in it, such as long indels comparable in scale to exons. Using only SNPs lets the authors magically take the asimulator transcripts from the simulation sample and plop them onto the linear reference assembly without worrying about coordinate liftover, but it could camouflage important flaws in the event definitions. So I don't think that this experiment actually manages to do what it is meant to do. Even given that pantas is using a basically-linear-reference-based approach embedded in a pangenome graph, it would still be good to make sure that it will not, for example, mistake a deletion of an exon or a splice site in the underlying sample haplotype for an instance of alternative splicing. Or, if it does have that shortcoming, it is important to know about it.

We agree with these observations and we thank the reviewer: we agree that it is important to state what our contribution can manage. However, since this is the first paper on alternative splicing detection from pangenome graphs, we did not have the ambition to solve all the issues raised. The reviewer highlighted two of these problems. First of all, to the best of our knowledge there is no simulator of RNA-Seq data based on pantranscriptomes that also supports the simulation of AS events. Since the goal of our paper is to present a tool for the classification and quantification of AS events, we decided to rely on state-of-the-art to simulate RNA-Seq reads (i.e., asimulator). The idea described by the reviewer is interesting and relevant, but we believe it is worth of a distinct investigation. Indeed, writing a simulator is not a simple task and it needs an ad-hoc methodology and an extensive experimental evaluation that, in our opinion, is a separate contribution/tool. This is a nice open problem: we pointed this

out in Section 3.1. However, in order to make the simulation more realistic, we used asimulator to simulate reads from both haplotypes of the selected sample. We still believe that including only SNPs in our simulation still provides a good evidence that AS event quantification can be performed on pantranscriptomes. Moreover, indels are included in the experiments on real data (both Drosophila and human). In our investigation we did not include structural variations. And this brings us to the second problem raised by the reviewer: how can we distinguish between alternative splicing events and structural variations? Unfortunately this is a very complex question and currently we do not have an answer. If we have a big deletion deleting a full exon only on one haplotype, can we be sure that this is not an exon skipping events with respect to some transcripts coming from the other haplotype? In this case, pantas will output an exon skipping event and we believe that this is the correct behaviour of our tool, since otherwise it will produce an incomplete output.
To distinguish between the two cases we need both RNA-Seq and DNA read, which is a fairly uncommon setting: that's why we focused on more common RNA-Seq datasets.
It is task of a downstream analysis to determine whether a call is due to an actual AS event or a different haplotype expressed. In any case, this problem is related to the task of analyzing the association between AS events and genomic variations. This requires an extensive analysis that goes beyond the goal of this paper.

Reviewer: Compared to the earlier parts of the paper, I think the experiments around quantification of alternative splicing are much stronger. But I didn't really understand the thread of reasoning about statistical significance. Are we testing the proposition that the true percent spliced in differs between two sets of sequencing reads, versus the null model that the true percent spliced in is the same? It seems odd to call these just "significant AS events" and not something like "significantly differential AS events", since as far as I can tell alternative splicing that isn't correlated with some particular variable under study is still alternative splicing. At one point it is said that "pantas reported 9 false significant events" and "pantas is able to retrieve more significant AS events without introducing more false calls", but the authors also say "pantas does not perform any statistical validation of its events yet", so it seems wrong to talk about it retrieving "significant" events. In general the use of the word "significant" in the paper needs to be carefully examined and restricted only to cases where a genuine statistical test was (correctly) performed.

Although we tried to use the same notation used in other papers (such as the SUPPA2 manuscript), we decided to replace all occurrences of "significant AS events" with "significantly differential AS events", as suggested by the reviewer. Moreover, we agree with the reviewer that we used the term "significant" too loosely. So we decided to reduce the number of occurrences of this term in order to avoid confusion. Finally, since `pantas` does not perform statistical validation, we heavily revised sections 3.2 and 3.3: we do not refer to `pantas` events as significant events anymore. We also clarified this at the start of Section 3:

> *We ran all tools with their default parameters and then, where indicated, we filtered the resulting set of AS events based on the reported statistical significance (as suggested by the tools' authors). However,*

*since **pantas** does not perform any statistical validation of its events yet, in all scenarios, we considered all the events reported without any post-filtering.*

Reviewer: Minor comments:
* The manuscript as a whole needs to be copy-edited for missing articles, number agreement, and similar minor grammatical issues.

We revised the entire manuscript and rewritten most of its part.

Reviewer: * The whole paper is in the context of short-read RNA-seq, but it refers just to "RNA-seq". Since long-read technologies can also sequence RNA, the paper should mention that it is targeting Illumina-scale reads, and also note the simulated read length distribution when discussing read simulation.

We would like to note that, in our opinion, `pantas` model does not depend on sequencing technology. However, the tool is currently limited to short-reads data. We made this clearer in the abstract.

Reviewer: * Formally, the linear reference walk R seems to be defined as a single walk and not a set of (disjoint) walks for different chromosomes. The formalism should consider multiple chromosomes, or the handwaving should be more explicit.

We pointed this out in our definition when introducing haplotype walks (we note that there is no more a reference walk R):

> *Each haplotype is the genomic sequence of a particular individual. We note that this sequence is usually a set of sequences (one per chromosome). However, without loss of generality, we will consider the haplotype walks as single walks in the graph.*

However, we believe that this is a simple generalization that does not impact the model since all events are defined with respect to haplotype-aware transcripts.

Reviewer: * The linear reference walk R is not constrained to be acyclic, nor is the graph as a whole, nor are the transcripts. Without this constraint, the reasoning in the definition of annotated exon skipping events, saying that a transcript that uses the junction <a, b> cannot also contain a and b with an exon between them, doesn't hold.

We explicitly state that `pantas` model works on acyclic graphs.

> *Although spliced pangenome can contain cycle, in this work we will focus on acyclic graphs.*

Extending `pantas` to cyclcic graph is now a future direction described in the conclusions.

Reviewer: * The haplotype set H does not appear to actually be used by pantas.

We fixed this. In the current version, we need the haplotypes since the haplotype-aware transcripts refer to them.

Reviewer: * Are "intronic" vertices always part of the linear reference? Or can a vertex be both an alternate allele and intronic (and therefore eligible to, say, participate in a novel cassette exon)?

The spliced pangenome is built from the reference plus a set of haplotypes. Therefore, an intronic vertex can be part of the reference walk or of any haplotype walk (if for instance it's an alternate allele). We made this clearer in the manuscript, Section 2.1:

> All other vertices represent intronic or intergenic regions that can belong to the reference walk as well as any haplotype walk.

Since intronic vertices are not annotated by pantas, any intronic vertex (reference or not) can partecipate in a novel event.

Reviewer: * The names used to refer to the different event types in the text on alternative splicing events are a different set of names than those used to refer to the events in the referenced panels of Figure 2 and Figure S3, which means the figure is of limited use for understanding the text.

We updated Figures 2 and S3 to keep consistency with the text.

Reviewer: * There is no support for mutually-exclusive exon events. If pantas wants to lay the groundwork for a fully-pangenomic treatment of alternative splicing, where different variants of exons might exist, it should at least think about mutually-exclusive exons when both are in the linear reference. Is this too hard because there's no single edge to focus on?

We agree that mutually exclusive events are very relevant. On the other hand, their formal characterization would even more complex than all those included in the paper, making the paper more challenging to absorb. In fact, as the reviewer pointed out, checking 2 or more junctions at the same time is necessary to call mutually-exclusive events. While the `pantas` model can be extended in that direction, we would prefer leave this as future direction.

Reviewer: * The definition given for how coverage is heuristically checked for a range of vertices claims to be assigning the set next(x_i) to the vertex x_i+1.

We thank the reviewer for pointing this out. We fixed that paragraph.

Reviewer: * Some definitions use concepts like two sets of exons being "consecutive" (as sets), which need a formal definition. I think I understand what they mean in the context of a particular transcript, but I'm filling in stuff that isn't on the page.

We fixed all definitions. Now consecutiveness is explicitly defined on the elements of the sets, that are the exons.

Reviewer: * The note about how checking conditions on each junction is "linear in the input size" isn't really justified. Some of those conditions in turn require doing set operations; I wouldn't describe something that comes out to O(# of junctions * # of exons), for example, as "linear in the input size".

We agree with the reviewer and we decided to remove that sentence. Since a thorough evaluation of the computational complexity of `pantas` is not the main goal of this paper, we think an extensive (and complex) analysis of its complexity would not provide substantial value.

Reviewer: * Why does pantas require read coverage on the focal edge e in order to detect annotated alternative splicing events? And then why in an asymmetrical way? Given the way that only the skipping edge is required to have weight above a threshold, won't exon skipping alternative splicing events be called when there is support for only the skipping isoform, while no event would be called when all the reads support the isoform with the exon included, even if that version is more unusual? Why is the longer version less "alternative"?

We better stated how `pantas` works in Section 2.2:

> *Thus* `pantas` *analyzes two sets of junctions, one coming from the* canonical *transcript whereas the other from the* alternative *one. Event detection starts from the alternative junctions and compare them to the canonical ones.*

So, we do not have a version that is "less alternative" but we have an alternative version that is compared to the canonical one. In this settings, we believe that if only the canonical version is supported, no alternative events should be called since the "alternative" is not expressed.

Reviewer: * In general, how do you know which isoform is constitutive and which is alternative? In my work on pangenomics I try to get away from the similar notion of a reference and an alternate allele: the pangenome is a reference and all the alleles in it are equally normal. But here there's a polarity to the events. Does it just follow the names, so that e.g. in an "exon skipping" event, the version with the exon included is always the "constitutive" version, even if it has only ever been observed under very specific circumstances? How does the polarity of an event type relate to which version of the event pantas requires to have read coverage in order to detect it? Is it consistent or so some events need coverage on the constitutive isoform while others need it on the alternative isoform?

`pantas` considers one isoform as the canonical one and it uses it as some sort of a reference. Unfortunately, we are not aware of any clear definition of alternative splicing in the context of a pantranscriptome that could be considered "reference-free". In this work we adapted the AS events concepts used in the literature to graph structures and we formally defined how to detect AS events on a pantranscriptome. To do so, we decided to maintain the "classical" reference-based nomenclatures of events and translate it to graph structure for ease of reading.

Reviewer: * When pantas "surjects" splice junction edge positions back to linear reference coordinates, how does it ensure that all the novel splice junction edges involved in novel events are constrained to connect points on the linear reference, for which it can "simply" find coordinates? Is the vg mpmap mapper prevented from producing splice junction edges that connect points on other nodes? Are these excluded when the graph is augmented? Or are they included in the graph but excluded from participation in events by carefully defining the novel events to not use them?

In the updated version, `pantas` does not perform the surject step anymore. Thanks to haplotype-aware annotation, any annotated junction can be mapped to the reference genome. Regarding novel junctions, we decided to avoid a remapping step that, given the current nature of the problem, is not guaranteed to produce exact mapping and could easily yield an imprecise location. Instead of providing wrong coordinates, we preferred to limit ourselves to those coordinates we can confidently report. However, since all events reported by `pantas` comprise at least one annotated junction, this allows for a more comprehensive visual inspection of the genomic locus via external tools.

Reviewer: * Why doesn't pantas do any statistical testing for differential alternative splicing between conditions? Shouldn't it be made to do that before publication? If not, how would a user run a significance test themselves on a particular event they are interested in?

We think that this is a very interesting problem worth of a future investigation. Given the complexity of the classification task, we focused on formalizing a definition of the problem and experiment one possible solution. These results can then be used as basis for a rigorous work on the event quantification, once the classification is solved.

Reviewer: * In the asimulator read simulation, the fact that only SNPs are used is doing a lot of work, because it means that the coordinates of the gene annotations on the linear reference are automatically the right coordinates to use on the sample to simulate from, and that the coordinates asimulator spits out on the sample being simulated from can be directly used as coordinates on the linear reference. If this weren't the case, there would be a liftover step required to get reference annotations into the assembly to be simulated from, and a liftover step to get asimulator's customized gene models back to the linear reference. The fact that the choice to use only SNPs is bypassing all this work is not explained, and indeed the fact that only SNPs are used from the DGRP is not mentioned in the main text.

The detailed answer is in a comment in page 12. In short, testing on more complex variants requires the development of new simulators. In our opinion, this would be a completely different tools. We now explicitly state in the main manuscript that we used only SNPs in the simulation.

Reviewer: * The authors note that they were not able to run an augmentation step successfully for whippet. Why not? What did the corresponding authors of whippet say when they complained? If they truly couldn't get it to work, they should report what they tried that was supposed to work but didn't in the supplement, in sufficient detail that other researchers can replicate the failure.

We opened an issue on the whippet github repository (`https://github.com/timbitz/Whippet.jl/issues/151`) but we did not receive any answer from the authors of whippet. We just followed the README and the tool crashed. We added more info in the main manuscript:

> On the other hand, `whippet` allows for augmenting the Contiguous Splice Graph with novel splice sites supported by read alignments, but we did not manage to run this augmentation successfully: `whippet` crashed when we asked to include novel splice sites from a BAM file. Without novel splice sites, `whippet` has been able to detect only novel exon skipping events, hence we decided to not include it in this analysis.

Reviewer: * In the ageing analysis in section 3.2, the authors write that, when genes overlap, an AS event can be supported "by reads not directly originating from that gene". I think this way of phrasing it is confusing; what's going on here is that which transcription start (or end?) site is picked can influence what the splicing machinery does elsewhere in the molecule, right? So the splicing that happens in transcripts that cover the full overlapping region can differ from the splicing that happens in transcripts that start or end in the middle, or that include the same shared sequence but different sequence before or after it. And can't a single original transcript get chopped up into multiple final mRNAs? I feel like this idea should be expressed more in terms of the missing context around the read, versus making it sound like the machinery set out to produce one gene's mRNA and not another, and knew the whole time which would end up being produced.

We revised the paragraph about overlapping genes as follows:

> *Indeed, overlapping genes increase the noise in the output of all tools and add further complexity in the AS quantification step since usually short RNA-Seq alignments in these regions is not sufficient to discern the different transcripts (coming from different genes) expressed in the dataset.*

Reviewer: * Also in 3.2, the authors note that aligning a single replicate took over 3 days, and as a result "any" analysis was "unfeasible" (maybe meant to be "infeasible"?). How many replicates would need to be analyzed? What is the compute budget that would need to be met to make the analysis feasible? Also, what is then being analyzed in the rest of this section, and how is it different from what was originally intended?

We replaced "unfeasible" with "infeasible". Our original plan was to align and analyze the entire RNA-Seq dataset. Since we were not interested in a comprehensive biological analysis of that dataset, but we wanted to compare the events quantification with those of state-of-the-art, we decided to subsample the datasets and we ran all the tools on this reduced set of reads (in order to have results that can be compared). Anyway, we would like to note that with a more powerful server it would not be necessary to perform this subsampling.

Reviewer: * Still in 3.2, the authors note at one point that they remove all events from consideration with an absolute delta PSI outside of 0.05 to 0.95, to remove the events "without a [sic] strong evidence of differential change". Why is this range bounded at

the top at all? Wouldn't an event with an absolute delta PSI of 0.96 have even stronger evidence of differential change than one at 0.94?

We thank the reviewer for pointing this out. We updated all the analyses keeping all events with $\Delta\psi \geq 0.05$.

Reviewer: * When kicking out "events reported as non statistically significant by the tool" at a common p-value threshold of 0.05, is the same regimen of multiple testing correction, if any, used in each case?

We would like to point out that we do not compute p-values of significance, but we used those computed by the various tools (as is the case for rMATS and SUPPA2).

Reviewer: How do the sizes of the different shared event sets compare to what they would be, given the anticipated false discovery rates, if there were no true differential alternative splicing events?

We apologize, but we have not understood the intended meaning of this comment. If it still applies to the revised version, we would be happy to incorporate the suggestion, but we need a more detailed description.

Reviewer: * The authors write that "The correlation achieved by pantas and all other approaches based on read alignment proves that read alignment to spliced pangenome is effective and can be confidently used for AS event differential quantification, with the even larger advantage of not being negatively affected by allelic bias." I don't think this is true; correlation between read-alignment-based tools proves that they are all doing similar or somehow isomorphic things as each other, not that the results are effective for any particular purpose (other than predicting what other read-alignment-based tools might output). It definitely doesn't prove anything about allelic bias. Consistency is necessary for the tools to all actually be getting at genuine differential alternative splicing events, but it isn't sufficient evidence to prove that that is what is happening.

We removed the last part of the sentence on allelic bias. Anyway, we still believe that the results show that `pantas` quantifications are reliable, since they are well correlated to `rMATS` quantification, that in our opinion is the best and most reliable state-of-the-art approach to perform AS event quantification.

Reviewer: * In the RT-PCR comparison in 3.3, the authors note that "aligning the 6 replicates required 1 week", and that therefore they "had" to design their gene-centric pipeline. It would be good here to specify what the compute budget was that they needed to achieve in order to actually run their analysis. If 1 week was too long, how long wouldn't be?

Our goal was not to extract any biological insight from the data, but we wanted to evaluate the quality of `pantas` results. We think that in a more biologically-oriented analysis, there should not be any time constraint.
However, in our settings we were more interested in evaluating the model. To address the reviewer concern, we removed that sentence from the manuscript. While rerunning the experiments, this time we had also an issue with the amount of RAM required to index the full graph (we pointed this out in Section 3.3).

Reviewer: * Still in 3.3, the set of true negatives seems to be a set of 44 exon-skipping events for which RT-PCR did not find a significant difference between the conditions. That's not really properly a true negative; the RT-PCR test necessarily has finite statistical power and might not detect a difference that does in fact exist between the conditions as significant. Given a p=0.05 threshold of significance, even if these events perfectly follow the null model, we should expect a re-test with RT-PCR to find 2.2 false positives, right? And if any of these events are RT-PCR false negatives but manage to be detected by other analyses, they would show up as false positives. I think the limitations of this approach to getting a truth set should be mentioned, and perhaps the whole experiment should be described in therms of "concordance" with RT-PCR, rather than true and false positives.

We agree with the reviewer that is not guaranteed to be a true negative, but we replicated the analysis done in the SUPPA2 paper and we used the same terminology (citing from the SUPPA2 paper: "Finally, using 44 RT-PCR negative cassette events that did not show any significant change upon the double knockdown of TRA2A and TRA2B, SUPPA2 had a lower false positive rate compared to the other methods"). Therefore we think that what we have done, while not perfect, is the current best practice.

Reviewer: The authors write that "25 events were reported as significant by all 4 tools", but pantas does not actually make a significance determination, so this can't be true. Do they mean to say that the events were "called" or otherwise generically asserted to genuinely be differentially alternatively spliced?

We agree with the reviewer. We removed the word "significant".

Reviewer: * The authors write that "A more polished statistical analysis of pantas results will allow to recover these events." How do they know that this will in fact happen if they haven't done it?

We agree with the reviewer. We changed "will allow" with "might allow".

Reviewer: * The phrase "all RT-PCR events were detected" probably was supposed to be something more like "all events were detected by RT-PCR".

We updated the sentence in the following way: "all RT-PCR validated events were detected". We wanted to say that all events that have been RT-PCR validated, have been detected by the tools.

Reviewer: And saying that this is "proving the complexity of correctly computing (and analyzing) the significance of differentially quantified AS events" doesn't mean anything to me and should probably be cut. Do they mean to say that determining whether alternative splicing is in fact differential or not is challenging? Or do they mean to imply that they think some of the tools tested are using incorrect statistics to compute their p-values or check them against their significance thresholds?

We thank the reviewer for pointing this out. We agree that the sentence is confusing and we removed it.

Reviewer: * The authors go on to talk about pantas reporting "9 false significant events", which makes no sense both because pantas doesn't actually test for statistical significance and because "false significance" isn't, as far as I know, a thing. You can incorrectly reject a null hypothesis that is actually true, and thus have a "false positive" in your set of supposedly-differential alternative splicing events. But that happens when your difference between your observed samples is truly a statistically significant difference, just one that happens to have occurred by chance. Or is "false" being used as shorthand for "false positive" here?

Following our revision of all occurrences of "significant", we removed this occurrence. Now `pantas` reports false events.

Reviewer: * How if at all does pantas distinguish between genuine alternative splicing and allele-specific changes in expression, when the two alleles have different gene structure? For example, a splice site may be absent in one copy of a gene, in cis with variation in its promoter region that causes its expression to change differently than that of the other copy in response to environmental conditions. Isoforms of the gene will change in frequency in response to environmental conditions, but not because of anything happening with the splicing machinery.

We thank the reviewer for pointing this case out. We are afraid that in this case, `pantas` would report the event since the current model and implementation consider haplotype-aware transcripts as independent transcripts with no relation between them. For this reason, events detected by `pantas` may "mix" transcripts coming from different haplotypes. Although this does not seem to impact the overall results of `pantas`, we still believe that this is an interesting case that needs a more thoughtful investigation. We added this in the Discussion section as future direction to be investigated.

Reviewer: * The authors in the conclusion lament that haplotype-aware splice junction/gene annotations are not yet available, and anticipate that they will benefit pantas when they become available, presumably because it will be able to be modified to support them. However, tools like the Comparative Annotation Toolkit exist today to propagate gene annotations between aligned haplotypes, and the Human Pangenome Reference Consortium's draft human pangenome reference includes CAT-based annotations for the haplotype assemblies involved. The requested annotations appear to already be available. Maybe they mean not available for Drosophila specifically?

We thank the reviewer for pointing this out. However, we wanted to provide a tool that is agnostic w.r.t. the organism. For this reason, we decided to not use the gene annotation released by the Human Pangenome Reference Consortium but we wrapped the construction of an annotated spliced pangenome in a Snakemake pipeline that can be used on any organism (starting from a reference, a gene annotation, and a catalog of population-level variations).

Reviewer: * The authors also write that, once haplotype-aware splice junction data becomes available, "pantas can be easily extended to associate AS events quantification [sic] to haplotype-specific variations". I'm not entirely sure if they really mean "haplotype-specific" (present on only a single haplotype?), or what "AS events quantification" is other than the process of counting how many times each version of an

alternative splicing event is observed, or exactly what it would mean to associate those two things. One reading might be that they will easily be able to use pantas to find genomic variants that effect the rate of alternative splicing, as quantitative trait loci. But I think they really mean to say that they will easily be able to upgrade pantas to a more fully pangenome-based analysis method that actually uses haplotype and/or pantranscriptome information. But I don't think that the authors have done what they would need to do in this paper to conclude that either of these can be done "easily". If they want to claim that pantas will easily be able to incorporate haplotype information, they need to phrase their theoretical model in a way that is not so linear-reference-centric and that can accommodate off-linear-reference transcripts in the transcript set. If they claimed that they have the theory all worked out and they just need the actual annotations to fully implement it, they could say that it would be easy. But they don't, and then they immediately go on to talk about unanswered questions about which exons should be thought of as the same as each other and how hard that will be to figure out.

We revised our conclusions taking into account the suggestions from the reviewer. Current version of `pantas` is haplotype-aware since it uses haplotype-aware transcripts (so that future direction is not needed anymore). We better explained what we mean by "haplotype-specific" events. However, being able to associate variations to changes in splicing is still - in our opinion - a not so well investigated direction. However, this is a quite complex task that usually requires both genomic and RNA-Seq data. We believe that this goes out the scope of our paper, so we revised the conclusions to remove any (implicit) reference to it.
