# Supplementary material for: Differential quantification of alternative splicing events on spliced pangenome graphs
Source: PLoS Comput Biol. 2024 Dec 9;20(12):e1012665. doi: 10.1371/journal.pcbi.1012665 (PMC11658704; doi:10.1371/journal.pcbi.1012665)
Supplement: Attachment — Submitted filename: pantas-answers.pdf [file pcbi.1012665.s003.pdf]

Responses to Reviewers (II)

"Differential quantification of alternative splicing events on spliced pangenome graphs"

Reviewer #2

Reviewer: The authors have substantially revised both their software and their manuscript in light of the reviewers' comments. The pantas tool now implements a defensible version of what alternative splicing detection could be like in a pangenomic context. While it could still be improved with the addition of built-in statistical testing, the rewritten manuscript no longer abuses the word "significant" and more defensibly places adding statistical testing under future work. The new analysis of the simulation experiments, with its precision-recall curves, is noticeably easier to understand than the previous tables, and the discussion of read simulation makes it much more clear that ignoring indels in the simulation is a load-bearing part of the procedure. The revised discussion of the RT-PCR concordance analysis also makes more sense.

Reviewer: My only remaining major concern is the way that the authors filtered events from the truth set in the simulated-data analysis for Figure 3. It looks like the authors are taking the set of events produced by each tool, and the set of events in each filtered truth set, and calling any event produced by the tool but not in the *filtered* truth set a "false positive". So, when running pantas with its w parameter set to 3, on the filtered truth set filtered at ω=20, if pantas sees an event that asimulator actually simulated, that has a read support of 10, and detects and produces it in its output, it gets penalized for creating a "false positive", since the event did not have a read support of at least 20. I don't think that this is a reasonable way to do this analysis. I also think that this approach is going to over-represent the performance of pantas, when one of the conditions tested has pantas's w parameter equal to the truth set filtering parameter ω; in Figure 3 this is at w = ω = 3. In that condition, pantas has access to the information that no event with a read support of less than 3 should be emitted, and will suppress any events it thinks have less read support even if it thinks they exist. The other tools don't have access to the true minimum read support, and will produce events where they think the read support is less than 3, because they don't know such events are ineligible to be in the truth set even if they were actually simulated into the data. This will differentially increase the false positive rate of tools other than pantas.
The authors should instead take an approach more typical of precision-recall or ROC plot curves, and keep the truth set constant along the curve while thresholding the output of each tool at different levels. This is how variant calling evaluation tools like hap.py work, keeping the set of true variants constant and filtering the call set under evaluation at different quality/confidence thresholds. If all tools report some notion of the observed read support for an event, then the events from each tool could be

thresholded on that to produce the different points along each curve. Alternatively, a tool-specific measure of confidence could be used to get a curve for each tool.
An alternate approach might be to identify all the true positives, false positives, and false negatives between each tool's results and the full, unfiltered truth set. Then, for each minimum read support value ω, drop the true positives and false negatives involving truth set events with *true* read support less than ω. This would avoid penalizing tools' computed precision scores when the tools are "too good" at identifying true events with low support, while still penalizing them for identifying false events with low support, and it would not be necessary for each tool to report a confidence or observed read support value.
Either approach would avoid the circularity of having conditions where pantas has access to the true minimum allowed read support while other tools do not.

We thank the reviewer for pointing this out. However, it is not fully clear to us how `pantas` can have access to the true minimum allowed read support. We ran `pantas` with a hard threshold on the required support (3 or 5, depending on the test) that was independent from the support reported by `asimulator`. It is true that `pantas` accuracy increases when the value of this parameter matches the threshold used to filter the truth set, but we would like to point out that even when the two values do not match, `pantas` has been able to achieve competitive accuracy (or superior, when $w \geq$ true support). Since there may have been some confusion in this regard, we explicitly stated this in the manuscript.

> *Indeed, when the w parameter is properly set to match the expected coverage of the events,* `pantas` *achieved the best trade-off between precision and recall. However, even when this parameter does not match the expected coverage,* `pantas` *has proved to be still able to achieve competitive (if not superior) accuracy (especially for highly supported events).*

In any case, we agree with the reviewer that we were penalizing the precision of the tools. We therefore decided to recompute their accuracy as suggested by the reviewer. We opted for solution #2 since it was not easy to select a good threshold for the output of all tools. We updated the corresponding plots and tables (Fig. 3, S2, S3, S4 and Table S2, S3). Figure 1 reports the comparison between the previous results (with penalized precision) and the new results. It is clear that we were penalizing all tools precision: new results show the same recall as before with higher precision. We are now indeed dropping true positives and false negatives w.r.t. true support (as we were already doing) but not false positives (improving the precision of the tools).

Reviewer: * The manuscript still contains a lot of number agreement errors and other similar copy-editing problems; the editors should go over it closely to find and remove these.

We revised the manuscript and we fixed the typos, problems, and inaccuracies we found.

Reviewer: * The visual difference between italic w, the pantas parameter, and lower case omega, the truth set filtering threshold, is very slight. It might be good to rename one of these symbols, or typeset one of them in a different font.

We agree with the reviewer that w and ω are way too similar. We replaced all occurrences of ω (true expected coverage from `asimulator`) with $\mathcal{W}$.

Reviewer: * The author summary contains the text "Pange3PM ESTnome", which is presumably a typo.

Fixed, thanks.

Reviewer: * The authors write that "pantas can pave the way to next generation bioinformatic approaches for the accurate analysis of the relations between genetic variations and alternative splicing aberrations". This seems, firstly, too specific: pantas could be useful for analysing relationships between genetic variation and all forms of alternative splicing, not just "aberrations". It also sounds vaguely eugenic: if a person can have genetic variation that causes alternative splicing aberrations, it is easy to slip into thinking of those alleles as aberrant alleles and that person as a person whose genes are substandard. Given the existence of non-scientists eager to find scientific work that they can deliberately misconstrue as supporting racism, as described in e.g. https://www.nature.com/articles/d41586-022-03252-z, it is important to write defensively around these issues. It does seem like "aberrant splicing events" is a concept in the field, defined along the lines of "rare large alterations of splice isoform usage" in https://doi.org/10.1038/s41588-023-01373-3, but if the authors are only going to use the word once in the summary, without expanding on what they mean, it might be safest to choose a different word. Perhaps this could be just "between genetic variations and alternative splicing", or "between genetic variations and medically-relevant alternative splicing".

We thank the reviewer for pointing this out. It was not our intention to sound eugenic. We fixed as suggested by removing the term "aberrations".

Reviewer: * The text in Figure 1 is much too small to read when viewing the figure at a normal size on a normal screen.

Unfortunately, we could not find a way to increase the font size while keeping all the necessary information. We would like to note that the image is in format that can be zoomed without quality loss (e.g., blurry image). Therefore, we decided to keep the image as it is in order to not remove valuable information.

Reviewer: * The precision/recall plots in Figure 3 probably should be square, with equal scales on both axes, to avoid visually prioritizing either precision or recall over the other metric.

We fixed the plots as suggested.

Reviewer: * The Figure 3 legend for "True Support" is in ascending order of support threshold imposed on the truth set, and so starts with the condition where tools are expected to achieve the highest precision and proceeds to conditions where the expected precision is lower. But this is right to left along the X axis in the plots, and backward from how I want to read them. The legend for the point shapes should be flipped so that it describes the points in the order they are expected to appear left to right.

We thank the reviewer for pointing this out. However, we did not like the idea of reversing the legend labels. To solve the issue raised by the reviewer, we decided to flip the x/y axes of the plots. Now legends and points follow the "left-to-right order".

Reviewer: * None of the Venn diagrams (like Figure 4a) are scaled to visually show the number of items in each category. They would be more useful if they were. The authors might also consider using UpSet plots here instead of Venn diagrams, which could turn these into more visually readable bar charts.

Unfortunately, we did not find a way to scale the circles. We also tried to use an UpSet plot as suggested but result was not good in our opinion. See, for instance, Figure 2 (this is the UpSet plot corresponding to Figure4(a) in the manuscript). We prefer the Venn diagram and we decided to keep the Venn diagrams as they are.

Reviewer: * In section 3.2, "an increase in the number of events (Suppl. Fig. S6b and Suppl.Table S5)" should reference S6a, and "strong loss of correlation (Suppl. Fig. S6a)" should reference S6b; the panel letters seem to be swapped.

Thanks, we fixed the references.

Reviewer: * In section 3.3 the authors write that they built a spliced pangenome from "128 random Europen samples" from the 1000 Genomes Project. I think "Europen" was probably meant to be "European". But that still doesn't make sense: the 1000 Genomes data doesn't categorize any samples as "European". It *does* have an "EUR" superpopulation that the data collectors created, described on their data portal as "European Ancestry", but that superpopulation includes many samples not properly describable as "European" due to not being sampled in Europe, such as "Utah residents (CEPH) with Northern and Western European ancestry". There are also a number of European samples, such as those from "Indian Telugu in the U.K.", that were sampled in Europe but not assigned to the EUR superpopulation. If the authors really drew from samples annotated as belonging to the EUR superpopulation in the 1000 Genomes VCF, then they need to say that, because it is an importantly different thing to be assigned to that superpopulation than it is to be European. If they drew from all samples collected in Europe, they should say that more explicitly.

We thank the reviewer for pointing this out. We indeed used the EUR superpopulation. We made this clearer in the text:

> *We considered 128 random samples from the `EUR` superpopulation, arbitrarily chosen, i.e., 128 individuals of European ancestry [...]*

Reviewer: * The authors don't justify drawing from only whatever they consider to be "Europen" samples, rather than from the whole 1000 Genomes dataset. Why was this decision made? It seems like maybe they are hoping to ethnicity-match or ancestry-match the MDA-MB-231 cell line from their reference 6. But that sample isn't from a 1000 Genomes population (which would generally require something like 4 grandparents with very similar self-reported ancestry). It instead comes with the outdated and vague (see https://www.nature.com/articles/d41586-021-02288-x) descriptor of "Caucasian"

(see https://www.cellosaurus.org/CVCL_0062), and was apparently collected at M D Anderson in Texas, which isn't in Europe. Since a racial descriptor like this isn't useful for ruling out recent ancestry from places other than Europe, or from populations that would be placed in 1000 Genomes superpopulations other than EUR, what justifies the restriction on sampling? There doesn't seem to actually be enough known about the ancestry of the target sample to try and match it, or to justify assuming that restricting sampling like this will produce better and not worse results. Was this particular restriction on sampling empirically discovered to produce better results than unrestricted sampling? Is there an analysis of the cell line that the authors could cite that shows that it looks a lot like the EUR superpopulation as a whole, but also not specifically like one or a few of the individual populations in it (which would then be better sets to sample from)?

The choice of the (super)population was arbitrary. There was no clear motivation behind our choice, if not that of building a less complex graph (in order to reduce the complexity of indexing it). We explicitly stated that the choice was arbitrary in the updated version of the manuscript.

Reviewer: * For the 44 RT-PCR negative differential alternative splicing events, 2/44 significant hits is nearly exactly the expected number of false discoveries for a p=0.05 threshold, when presented with 44 candidates from the null model's distribution. It might make sense to note this when describing the results from for the tools that perform statistical significance testing; on their own terms they *shouldn't* be producing noticeably fewer false positives than that.

There was a typo in the manuscript: `whippet` was repeated twice and this may have created some confusion. Only `SUPPA2` reported 2 false events. Other tools reported more events (4 for `whippet` and 10 for `rMATS`). For this reason, we decided to not delve into conjecturing reasons behind these results.

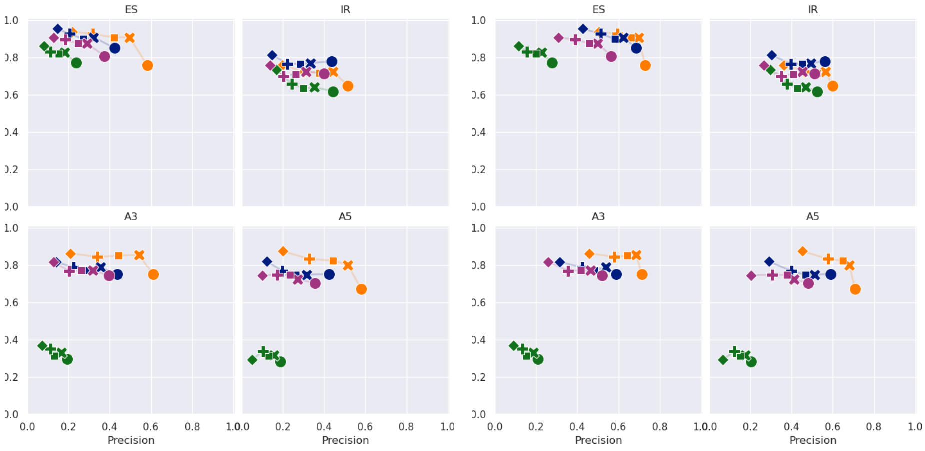

(a) (b)

Figure 1: (a) Old results with penalized precision. (b) New results following reviewer suggestion (n.2).

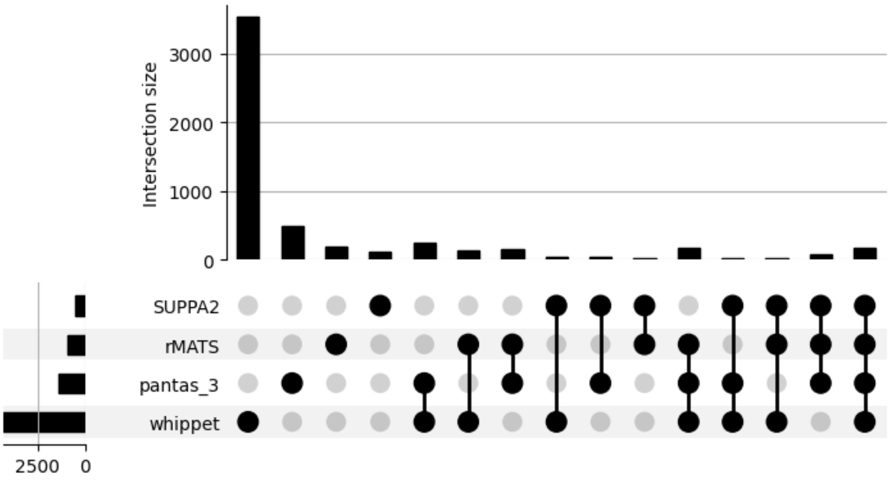

Figure 2: Upset plot
